# TXNIP mediates ferroptosis in a bronchopulmonary dysplasia mouse model by regulating the SLC7A11/GPX4 pathway

**DOI:** 10.1038/s41598-025-19092-6

**Published:** 2025-10-08

**Authors:** Dongzhui Chen, Feifei Yin, Pin Yang, Wanrong Xia, Yi Huang, Yue Feng, Li Yang, Shuqiang Lin, Qiuyue Zhang

**Affiliations:** 1 The First School of Clinical Medicine, Hainan Medical University, Haikou, 570102 Hainan China; 2 Hainan Medical University-The University of Hong Kong Joint Laboratory of Tropical Infectious Diseases, Key Laboratory of Tropical Translational Medicine of Ministry of Education, School of Basic Medicine and Life Sciences, Hainan Medical University, Haikou, 571199 Hainan China; 3 Department of Pediatrics, First Affiliated Hospital of Hainan Medical University, Haikou, 570102 Hainan China

**Keywords:** Bronchopulmonary dysplasia, Thioredoxin-interacting protein, Ferroptosis, Adenovirus serotype 5, Oxidative stress, Diseases, Medical research, Molecular medicine, Pathogenesis

## Abstract

Bronchopulmonary dysplasia (BPD) is one of the most common respiratory diseases in preterm infants. Studies have shown that ferroptosis is involved in the pathogenesis of BPD, and thioredoxin-interacting protein (TXNIP)-mediated oxidative stress also plays a role in its development. The solute carrier family 7 member 11 (SLC7A11)/glutathione peroxidase 4 (GPX4) signaling pathway is one of the key pathways regulating ferroptosis. This study aimed to investigate the mechanism by which TXNIP regulates SLC7A11/GPX4 pathway-mediated ferroptosis in BPD. Mice were randomly divided into ten treatment groups: Air, Hyperoxia, Hyperoxia + RSL3, Hyperoxia + Ferrostatin-1, Hyperoxia + Ad-shNC, Hyperoxia + Ad-shTXNIP, Hyperoxia + Ad-vector, Hyperoxia + Ad-TXNIP, Hyperoxia + Ad-shSLC7A11, and Hyperoxia + Ad-shTXNIP + Ad-shSLC7A11. Lung tissues were collected for hematoxylin and eosin (H&E) staining to observe pathological changes, and transmission electron microscopy (TEM) was used to examine mitochondrial morphology. Real-time quantitative polymerase chain reaction (qPCR) was performed to measure the mRNA expression of TXNIP, SLC7A11, and GPX4. Western blotting was used to analyze the protein levels of TXNIP, SLC7A11, and GPX4. GPX4 activity was determined using an enzyme-linked immunosorbent assay (ELISA). Additionally, glutathione (GSH), malondialdehyde (MDA), and ferrous iron (Fe^2+^) levels in lung tissues were measured using corresponding detection kits. H&E staining and TEM confirmed successful modeling of lung injury after hyperoxia. MDA and Fe^2+^ increased, GSH decreased, and GPX4 activity declined after hyperoxia. TXNIP mRNA and protein were upregulated by hyperoxia, while SLC7A11 and GPX4 were downregulated. Ferroptosis activation exacerbated lung injury, whereas inhibition alleviated it. TXNIP overexpression using adenovirus serotype 5 (Ad5) aggravated ferroptosis in BPD, and this was ameliorated by TXNIP knockdown. Knockdown of the upstream signal SLC7A11 increased ferroptosis in BPD, and simultaneous knockdown of TXNIP and SLC7A11 reversed the ferroptosis reduction. In conclusion, in the BPD model, TXNIP expression was increased, and TXNIP promoted ferroptosis by downregulating the SLC7A11/GPX4 pathway. These findings suggest that gene therapy targeting TXNIP knockdown using adenovirus as a vector may serve as a therapeutic strategy for BPD.

**Supplementary Information:**

The online version contains supplementary material available at 10.1038/s41598-025-19092-6.

## Introduction

Bronchopulmonary dysplasia (BPD), also known as chronic lung disease, is one of the most common respiratory disorders in preterm infants. It was first reported and named in 1967 by Northway, Rosen, and Porter^1^. With the rapid advancement of medical technology in recent years, the survival rate of preterm infants has significantly improved; however, the incidence of BPD has risen from 20.8% in 2010 to 40.7% in 2019, showing an annual upward trend^2,3^. BPD is closely associated with post-neonatal mortality, rehospitalization rates, childhood health status, and neurodevelopmental outcomes, imposing substantial economic and psychological burdens on families and society, and severely affecting the quality of life of affected children^4^. Currently, the pathogenesis of BPD remains incompletely understood, and there are no effective preventive or therapeutic measures available in clinical practice^5–7^.

Ferroptosis, a novel form of programmed cell death^8^, has been shown to be closely related to BPD^9–12^. The solute carrier family 7 member 11 (SLC7A11)/glutathione peroxidase 4 (GPX4) signaling pathway, an antioxidant pathway involved in amino acid metabolism, plays a critical role in ferroptosis. Inhibition of the cystine/glutamate antiporter (also known as system xc-) leads to insufficient synthesis of glutathione (GSH), which in turn suppresses the activity of GPX4. This significantly reduces the cell’s ability to resist lipid peroxidation, thereby inducing ferroptosis^13^. Studies have also demonstrated that modulation of the SLC7A11/GPX4 pathway regulates ferroptosis^14,15^.

Thioredoxin-interacting protein (TXNIP) was first identified in 1994 in HL-60 promyelocytic leukemia cells treated with 1,25-dihydroxyvitamin D_3_ (1,25-(OH)_2_D_3_) and was also named vitamin D_3_ up-regulated protein 1 (VDUP1)^16^. TXNIP exhibits various biological functions, including regulation of oxidative stress, as well as modulation of inflammatory responses, autophagy, and apoptosis^17–20^. In recent years, TXNIP-mediated oxidative stress has been shown to play significant roles in cancer, diabetes, cardiovascular diseases, and neurodegenerative disorders^21–23^. Research has revealed that alterations in TXNIP expression may affect the expression of vascular endothelial growth factor (VEGF) in infant lungs, suggesting that TXNIP is involved in the pathogenesis of BPD^24,25^. Recent studies have further indicated that ubiquitin-like containing PHD and RING finger domains 1 (UHRF1) overexpression promotes PTEN-induced kinase 1 (PINK1)-mediated mitophagy and suppresses TXNIP expression, while upregulating GPX4 and SLC7A11 expression. This suggests a potential correlation between TXNIP and expression of GPX4 and SLC7A11^26^ . However, whether TXNIP participates in ferroptosis in BPD and whether it mediates ferroptosis through regulation of the SLC7A11/GPX4 pathway remains to be further explored.

In this study, we investigated how TXNIP regulates ferroptosis in BPD through the SLC7A11/GPX4 pathway, aiming to identify potential genetic targets for the prevention and treatment of BPD.

## Results

### Schematic representation of the experimental design

**Fig. 1:**
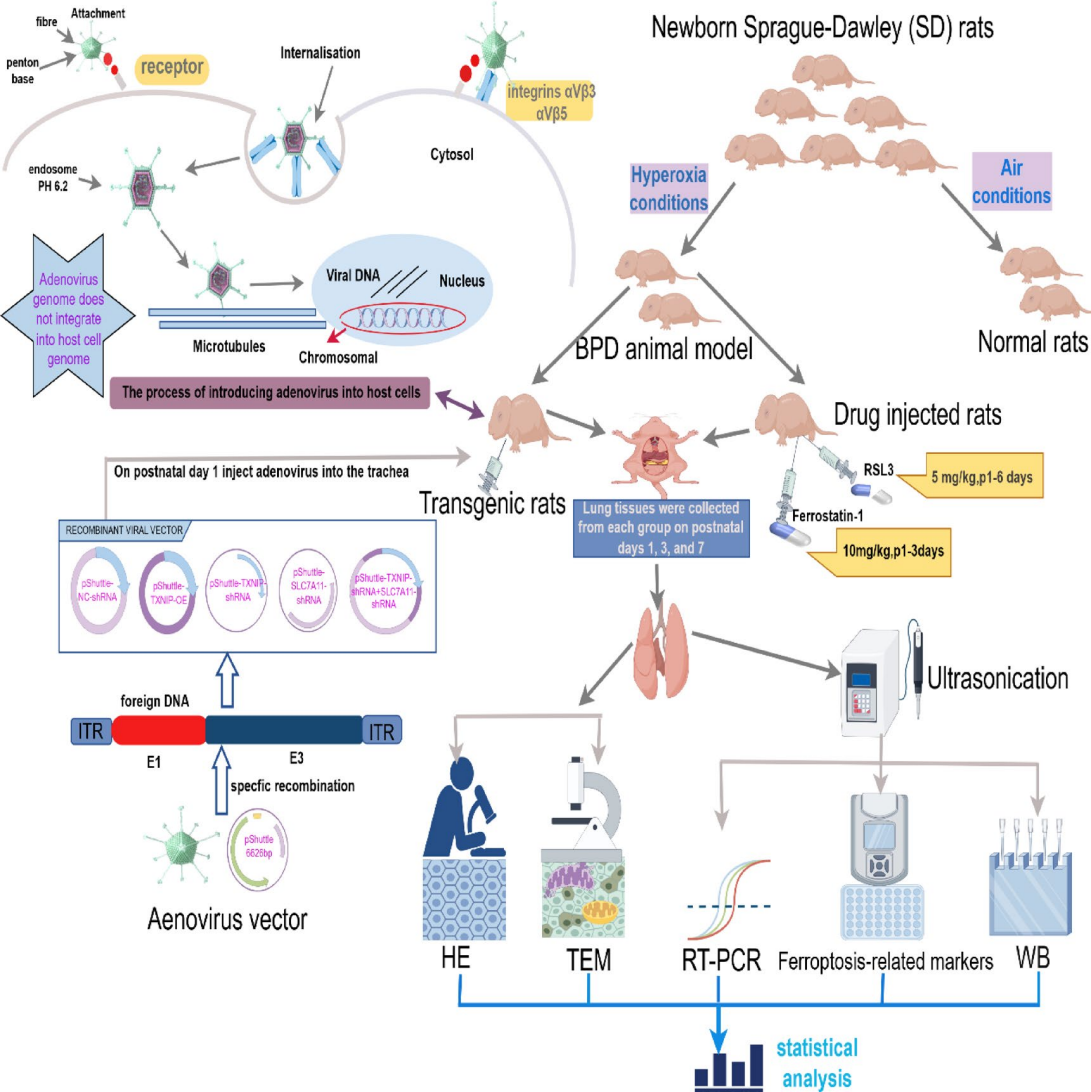
Schematic representation of the experimental design. ITR, inverted terminal repeat; E1, early region 1‌; E3, early region 3; HE, hematoxylin and eosin (H&E) staining; TEM, transmission electron microscopy; RT-PCR, reverse transcription quantitative polymerase chain reaction; WB, western blot; BPD, bronchopulmonary dysplasia.

As shown in Fig. 1, neonatal rats were exposed to hyperoxic conditions to establish a model of BPD. Animals were divided into groups and given various treatments, as follows: ferroptosis modulation: rats were intraperitoneally injected with 10 mg/kg of the ferroptosis inhibitor Ferrostatin-1 daily for 3 days or injected with 5 mg/kg of the ferroptosis activator RSL3 daily for 6 days. Adenovirus intervention genes: Adenovirus serotype 5 (Ad5) vectors for overexpression of TXNIP and knockdown of TXNIP and SLC7A11 were constructed through in vitro transcription. On postnatal day 1, rats in the overexpression and knockdown groups were anesthetized, and a cervical incision was made to expose the trachea. A 30-gauge needle was used to inject 100 μL of the corresponding adenovirus into the trachea, with an adenovirus titer of 1.8 × 10^8^ pfu. Finally, samples were collected to complete the endpoint experiment: lung tissues were harvested on P1, P3, and P7 for histological analysis (hematoxylin and eosin staining (H&E), transmission electron microscopy (TEM)), quantitative analysis of ferroptosis-related markers (Fe^2+^, MDA, GSH, GPX4), RT-qPCR and western blotting. The purpose of this experiment was to evaluate ferroptosis activation in hyperoxia-induced BPD and to elucidate the mechanism underlying TXNIP regulation of SLC7A11/GPX4 pathway-mediated ferroptosis in BPD.

### Increased expression of TXNIP in the high oxygen-induced BPD animal model

**Fig. 2:**
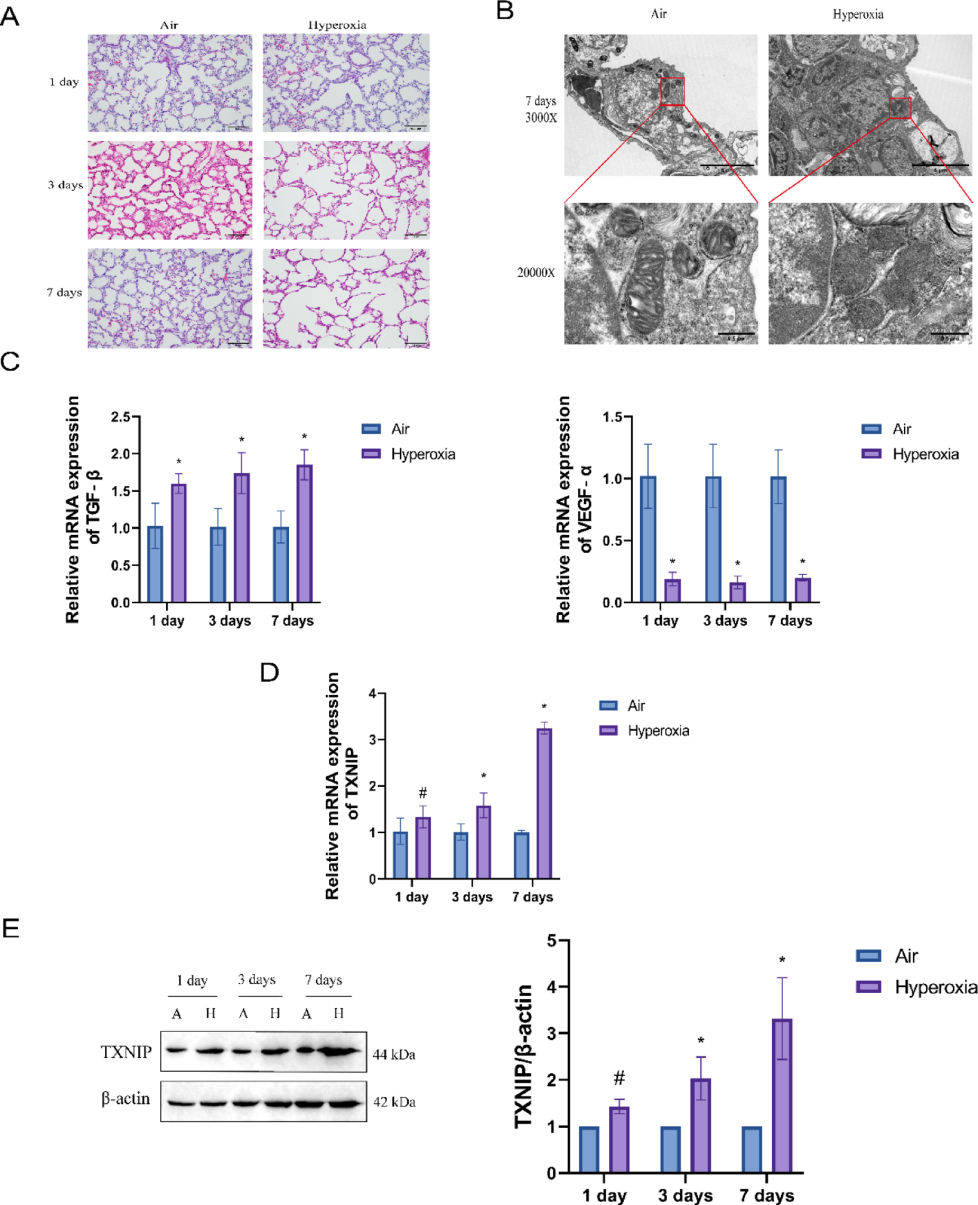
Validation of successful establishment of a neonatal rat model and expression levels of thioredoxin-interacting protein (TXNIP) in the bronchopulmonary dysplasia (BPD) model. (**A**) H&E staining was used to assess lung damage in the Air and Hyperoxia groups on postnatal days 1, 3, and 7. (**B**) TEM revealed ultrastructural changes in lung cells in the Air and Hyperoxia groups on postnatal day 7. (**C**) RT-qPCR analysis of mRNA expression of TGF-β and VEGF-α genes in the Air and Hyperoxia groups on postnatal days 1, 3, and 7. (**D**) RT-qPCR analysis of TXNIP mRNA expression in the Air and Hyperoxia groups on postnatal days 1, 3, and 7. (**E**) Western blot analysis of TXNIP protein expression in lung tissues in the Air and Hyperoxia groups on postnatal days 1, 3, and 7 (original WB images are presented in Supplementary information). Data are presented as mean ± SD (n = 6), and were analyzed by two-way analysis of variance (ANOVA) with Tukey’s post hoc test for inter-group multiple comparisons; #* P* > 0.05 vs. Air; * P < 0.05 vs. Air.

To investigate the expression of TXNIP in a BPD model, we first established an animal model of BPD using hyperoxia. The H&E staining results, as shown in Fig. 2A, revealed that the alveolar structure of the lung tissue in the Air group was uniform, with clear and evenly-distributed cytoplasm and nuclei. In contrast, the Hyperoxia group exhibited thinner alveolar walls, uneven alveolar sizes, partial alveolar fusion, and a significant reduction in the number of alveoli, along with a trend towards structural simplification. Additionally, some pulmonary septa showed significant thickening, with vascular dilation, interstitial hyperplasia, and fibrotic proliferation. TEM results, as depicted in Fig. 2B, demonstrated that mitochondria in the lung tissue of the Air group were intact, with a double-membrane structure and clearly visible cristae. In contrast, the mitochondria in the Hyperoxia group were severely damaged, showing shrinkage, vacuolization, and disappearance of cristae. RT-qPCR analysis of TGF-β and VEGF-α mRNA expression, as illustrated in Fig. 2C, indicated that, compared to the Air group, the Hyperoxia group had decreased VEGF-α mRNA expression and increased TGF-β mRNA expression. These findings suggest that the BPD model was successfully established in neonatal rats exposed to hyperoxia. Subsequently, RT-qPCR was used to measure TXNIP mRNA expression, as shown in Fig. 2D. The results indicated that the longer the exposure to hyperoxia, the more significant the increase in TXNIP mRNA expression compared to the Air group. Western blot analysis of TXNIP protein expression levels in the lung tissues of the two groups, as presented in Fig. 2E, showed the same effect. These results demonstrate that TXNIP expression is increased in neonatal rats in a hyperoxia-induced BPD model.

### Ferroptosis occurs in high oxygen-induced BPD

**Fig. 3:**
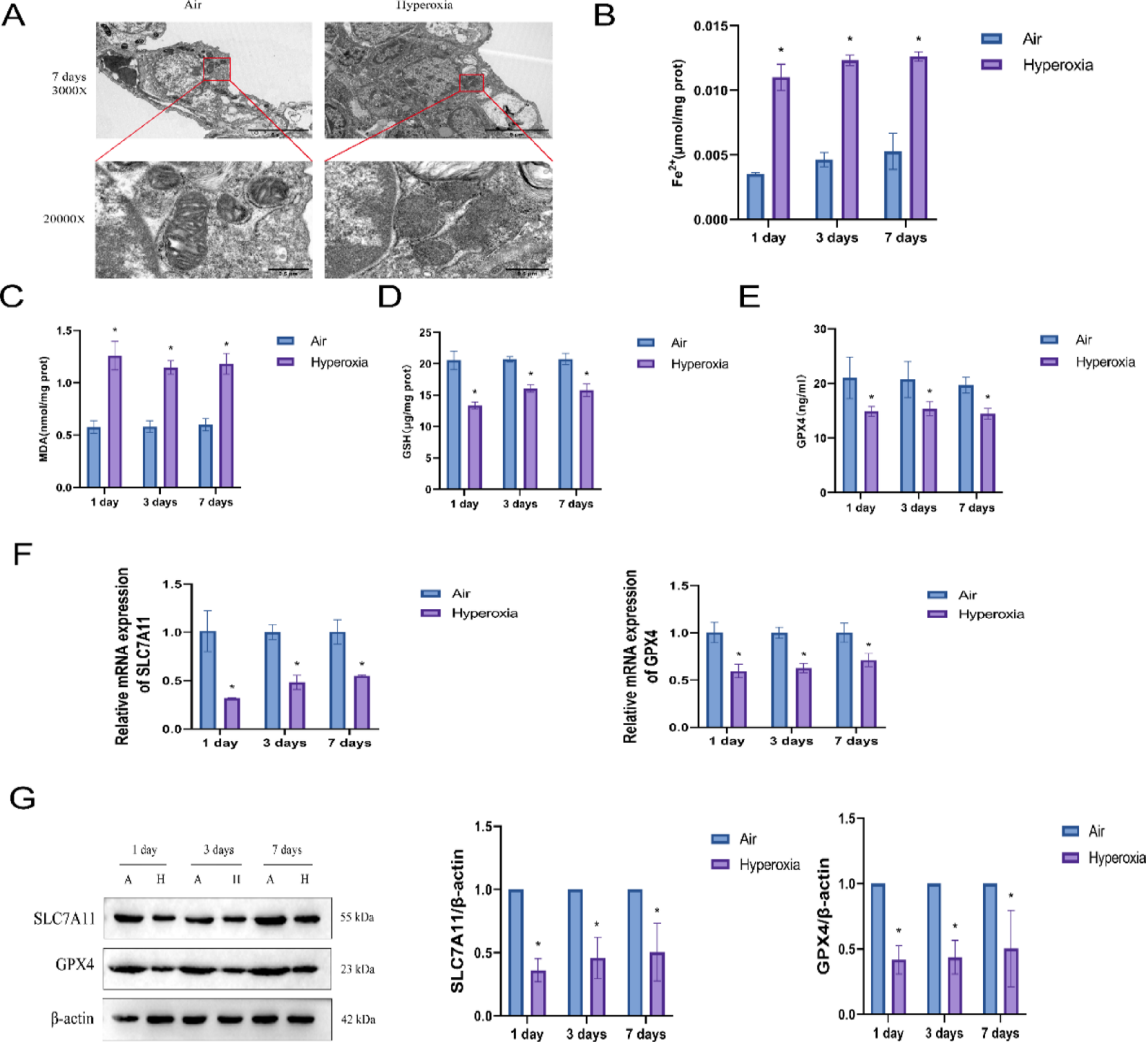
Ferroptosis and related pathway expression in hyperoxia-induced BPD. (**A**) TEM assessment of ultrastructural changes in lung tissue in the Air and Hyperoxia groups on postnatal day 7. (**B**) Measurement of Fe^2+^ levels using an iron assay kit in the Air and Hyperoxia groups on postnatal days 1, 3, and 7. (**C**) Measurement of MDA levels using a malondialdehyde (MDA) assay kit in the Air and Hyperoxia groups on postnatal days 1, 3, and 7. (**D**) Measurement of glutathione (GSH) levels using a GSH assay kit in the Air and Hyperoxia groups on postnatal days 1, 3, and 7. (**E**) Enzyme-linked immunosorbent assay (ELISA) detection of glutathione peroxidase 4 (GPX4) activity in the Air and Hyperoxia groups on postnatal days 1, 3, and 7. (**F**) RT-qPCR analysis of SLC7A11 and GPX4 mRNA expression in the Air and Hyperoxia groups on postnatal days 1, 3, and 7. (**G**) Western blot analysis of SLC7A11 and GPX4 protein expression in the Air and Hyperoxia groups on postnatal days 1, 3, and 7 (original WB images are presented in Supplementary information). Data are presented as mean ± SD (n = 6); data were analyzed by two-way analysis of variance (ANOVA) with Tukey’s post hoc test for inter-group multiple comparisons; * P < 0.05 vs. Air.

To confirm the presence of ferroptosis and its underlying mechanisms in BPD model mice, we first examined the lung tissues by TEM to observe mitochondrial morphology. As shown in Fig. 3A, mitochondria in the lung tissues of the Air group exhibited an intact structure, with a double membrane and clearly visible cristae. In contrast, mitochondria in the Hyperoxia group showed severe structural damage, including shrinkage, vacuolization, and loss of cristae, which are characteristic features of ferroptosis. We then measured ferrous iron levels in lung tissues. As shown in Fig. 3B, compared with the Air group, the Hyperoxia group exhibited increased Fe^2+^ content. Similarly, we measured MDA levels in lung tissues. As shown in Fig. 3C, compared with the Air group, the Hyperoxia group exhibited elevated MDA content, indicating increased lipid peroxidation. Additionally, we measured GSH levels in lung tissues. As shown in Fig. 3D, compared with the Air group, the Hyperoxia group exhibited decreased GSH content. We also measured the activity of GPX4 using an enzyme-linked immunosorbent assay (ELISA). As shown in Fig. 3E, compared with the Air group, the Hyperoxia group exhibited significantly reduced GPX4 activity. We further investigated the mRNA expression of SLC7A11 and GPX4 genes using RT-qPCR. As shown in Fig. 3F, compared with the Air group, the Hyperoxia group exhibited reduced mRNA expression of both SLC7A11 and GPX4. Finally, we performed western blot analysis to examine the protein expression of SLC7A11 and GPX4 (Fig. 3G), and this confirmed that protein expression of both SLC7A11 and GPX4 was decreased in the Hyperoxia group. These results indicate that ferroptosis occurs in hyperoxia-induced BPD and downregulates expression of SLC7A11 and GPX4 genes.

### Regulation of ferroptosis modulates high oxygen lung injury

**Fig. 4:**
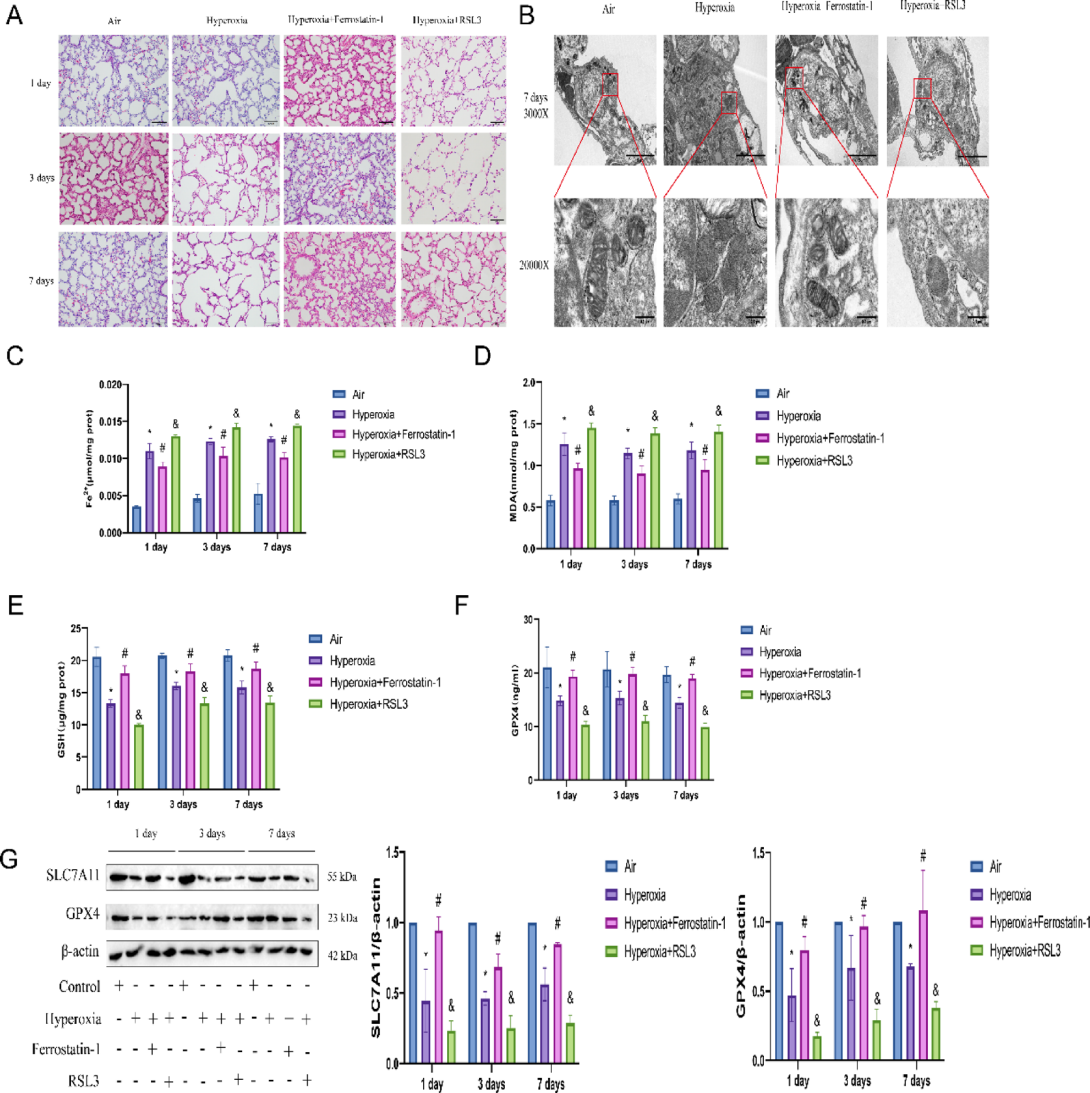
Lung tissue condition after modulation of ferroptosis. (**A**) H&E staining of lung tissue to assess pathological changes in lung injury in the Air, Hyperoxia, Hyperoxia + Ferrostatin-1 and Hyperoxia + RSL3 groups on postnatal days 1, 3, and 7. (**B**) TEM assessment of ultrastructural changes in lung tissue in the Air, Hyperoxia, Hyperoxia + Ferrostatin-1 and Hyperoxia + RSL3 groups on postnatal day 7. (**C**–**F**) Quantitative analysis of ferroptosis-related markers in lung tissues from the Air, Hyperoxia, Hyperoxia + Ferrostatin-1 and Hyperoxia + RSL3 groups on postnatal days 1, 3, and 7. (**C**) Measurement of Fe^2+^ levels using an iron assay kit. (**D**) Measurement of MDA levels using an MDA assay kit. (**E**) Measurement of GSH levels using a GSH assay kit. (**F**) Measurement of GPX4 activity using a GPX4 assay kit. (**G**) Western blot analysis of SLC7A11 and GPX4 protein expression in lung tissue in the Air, Hyperoxia, Hyperoxia + Ferrostatin-1 and Hyperoxia + RSL3 groups on postnatal days 1, 3, and 7. In the Hyperoxia + Ferrostatin-1 group, rats were intraperitoneally injected with 10 mg/kg of the ferroptosis inhibitor Ferrostatin-1 daily for 3 days. In the Hyperoxia + RSL3 group, rats were intraperitoneally injected with 5 mg/kg of the ferroptosis activator RSL3 daily for 6 days. (original WB images are presented in Supplementary information). Data are presented as mean ± SD (n = 6); analysis was performed using two-way analysis of variance (ANOVA) with Tukey’s post hoc test for inter group multiple comparisons; * P < 0.05 vs. Air; #* P* < 0.05 vs. Hyperoxia; &* P* < 0.05 vs. Hyperoxia.

To further investigate the activation of ferroptosis in BPD, we treated rats in the BPD model group with ferroptosis inducers and inhibitors, and performed H&E staining. As shown in Fig. 4A, compared with the hyperoxia group, the number of alveoli increased and the thickness of alveolar septa decreased in the ferroptosis inhibitor group. However, the addition of a ferroptosis inducer reduced the alveolar count again and simplified the structure. Additionally, we examined the ultrastructure of lung tissues using TEM. As shown in Fig. 4B, the mitochondrial structure in samples from the hyperoxia group was severely disrupted, with shrinkage, vacuolization, and loss of cristae. Compared with the hyperoxia group, the degree of mitochondrial damage was alleviated in the ferroptosis inhibitor group, with some mitochondria retaining a double-membrane structure and cristae, although some still exhibited vacuolization. However, the addition of a ferroptosis inducer severely disrupted the mitochondrial structure again, causing mitochondrial shrinkage, vacuolization, and loss of cristae. We also measured iron levels in lung tissues. As shown in Fig. 4C, compared with the hyperoxia group, the ferroptosis inducer group showed increased Fe^2+^ content, while the ferroptosis inhibitor group showed a decrease. Similarly, we measured MDA levels, and found that the MDA content was elevated in the ferroptosis inducer group, while it was reduced in the ferroptosis inhibitor group (Fig. 4D). Next, we measured glutathione (GSH) levels. As shown in Fig. 4E, the GSH content was decreased in the ferroptosis inducer group, while it was increased in the ferroptosis inhibitor group. We also performed an ELISA to analyze the activity of GPX4, which revealed that GPX4 activity was decreased in the ferroptosis inducer group, while it was increased in the ferroptosis inhibitor group (Fig. 4F). Finally, we analyzed protein levels using western blotting. As shown in Fig. 4G, the protein expression of SLC7A11 and GPX4 was decreased in the ferroptosis inducer group, but increased in the ferroptosis inhibitor group. These results indicate that modulating ferroptosis regulates hyperoxia-induced lung injury, and that promoting ferroptosis exacerbates BPD.

### Overexpression of TXNIP aggravates BPD in vivo

**Fig. 5:**
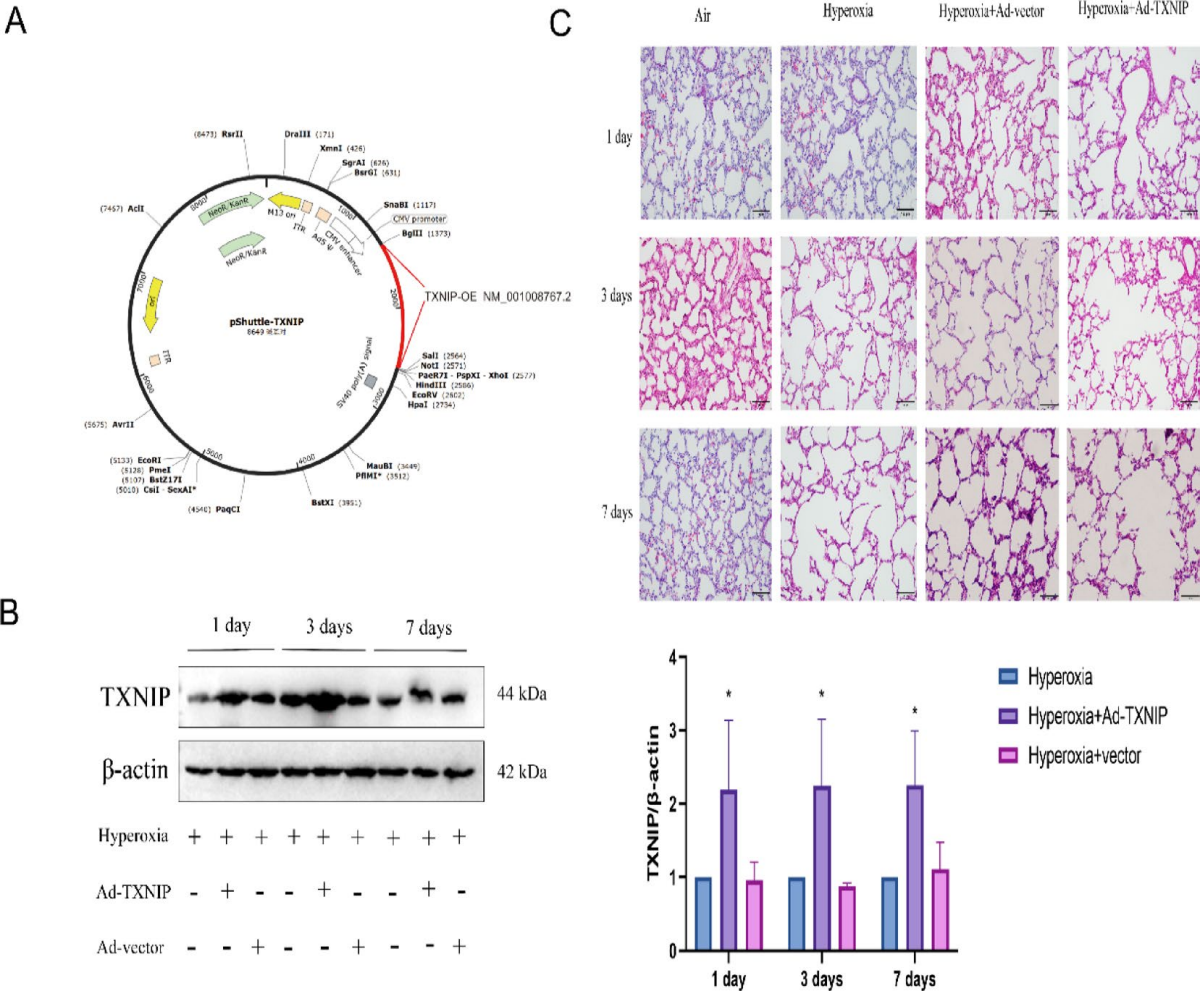
The condition of lung tissues after TXNIP overexpression. (**A**) Schematic diagram of the lentiviral vector for TXNIP overexpression. (**B**) Western blot analysis of TXNIP protein expression in lung tissues in the Hyperoxia, Hyperoxia + Ad-vector and Hyperoxia + Ad-TXNIP groups on postnatal days 1, 3, and 7 (original WB images are presented in Supplementary information). (**C**) H&E staining of lung tissues to assess pathological changes in lung injury in the Air, Hyperoxia, Hyperoxia + Ad-vector and Hyperoxia + Ad-TXNIP groups on postnatal days 1, 3, and 7. In the adenovirus rat group, Adenovirus serotype 5 (Ad5) vectors for overexpression of TXNIP were constructed through in vitro transcription .On postnatal day 1, rats in the overexpression groups were anesthetized, and a cervical incision was made to expose the trachea. A 30-gauge needle was used to inject 100 μL of the corresponding adenovirus into the trachea, with an adenovirus titer of 1.8 × 10^8^ pfu. Data are presented as mean ± SD (n = 6); two-way analysis of variance (ANOVA) was used, combined with Tukey’s post hoc test for inter group multiple comparisons; * *P* < 0.05 vs. Hyperoxia.

To investigate the impact of TXNIP on BPD, we utilized an adenoviral vector overexpressing the TXNIP gene for in vitro transcription to construct TXNIP-overexpressing rats. The structure of the TXNIP overexpression adenoviral vector is shown in Fig. 5A. After in vivo transcription and expression for 1, 3, or 7 days, lung tissues were collected from the rats for western blot analysis. As shown in Fig. 5B, TXNIP protein expression in the Hyperoxia + Ad-TXNIP group was significantly higher than that in either the Hyperoxia or Hyperoxia + Ad-vector groups, indicating successful overexpression of TXNIP in the transgenic rats. Subsequently, H&E staining was performed. As shown in Fig. 5C, the lung tissues of Air-exposed neonatal rats exhibited uniform alveolar structures with clear cytoplasm and nuclei, and evenly distributed cells. Compared with the Air group, the Hyperoxia and Hyperoxia + Ad-vector groups showed thinning of the alveolar walls, uneven alveolar sizes, fusion of some alveolar spaces, a significant reduction in alveolar numbers, and simplification of alveolar structures. Additionally, some pulmonary septa were markedly thickened, with evidence of vascular dilation, interstitial proliferation, and fibrosis. Compared with the Hyperoxia and Hyperoxia + Ad-vector groups, overexpression of TXNIP exacerbated the pathological changes in lung tissues induced by hyperoxia. The Hyperoxia + Ad-TXNIP group exhibited more pronounced thinning of alveolar walls, more uneven alveolar sizes, more obvious fusion of alveolar spaces, a more significant reduction in alveolar numbers, and greater structural simplification. These results indicate that overexpression of TXNIP exacerbates BPD.

### Overexpression of TXNIP in vivo promotes ferroptosis in BPD

**Fig. 6:**
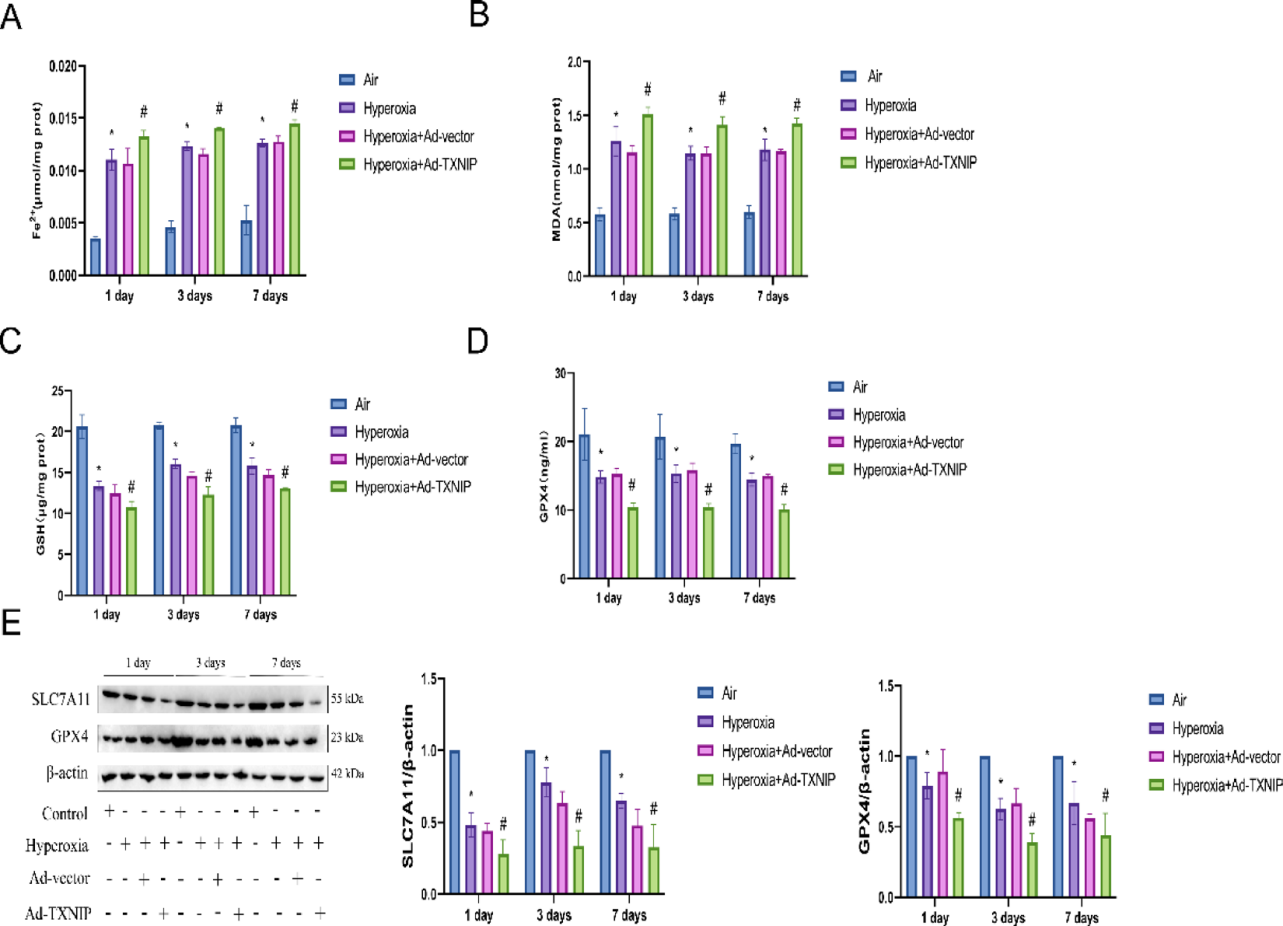
Ferroptosis status following TXNIP overexpression in the BPD model. (**A**–**D**) Quantitative analysis of ferroptosis-related markers in lung tissues from the Air, Hyperoxia, Hyperoxia + Ad-vector and Hyperoxia + Ad-TXNIP groups on postnatal days 1, 3, and 7. (**A**) Measurement of Fe^2+^ levels using an iron assay kit. (**B**) Measurement of MDA levels using an MDA assay kit. (**C**) Measurement of glutathione (GSH) levels using a GSH assay kit. (**D**) Measurement of GPX4 activity using a GPX4 assay kit. (**E**) Western blot analysis of SLC7A11 and GPX4 protein expression in lung tissue in the Air, Hyperoxia, Hyperoxia + Ad-vector and Hyperoxia + Ad-TXNIP groups on postnatal days 1, 3, and 7 (original WB images are presented in Supplementary information). In the adenovirus rat group, Adenovirus serotype 5 (Ad5) vectors for overexpression of TXNIP were constructed through in vitro transcription. On postnatal day 1, rats in the overexpression groups were anesthetized, and a cervical incision was made to expose the trachea. A 30-gauge needle was used to inject 100 μL of the corresponding adenovirus into the trachea, with an adenovirus titer of 1.8 × 10^8^ pfu. Data are presented as mean ± SD (n = 6); two-way analysis of variance (ANOVA) followed by Tukey’s post hoc test was used for inter-group multiple comparisons; * *P* < 0.05 vs. Air; # *P* < 0.05 vs. Hyperoxia.

To assess the relationship between TXNIP and ferroptosis in BPD, we overexpressed TXNIP in rats and examined the biomarkers of ferroptosis. As shown in Fig. 6A, compared with the Air, Hyperoxia, and Hyperoxia + Ad-vector groups, the Hyperoxia + Ad-TXNIP group exhibited significantly elevated levels of ferrous iron (Fe^2+^). Similarly, the Hyperoxia + Ad-TXNIP group showed increased levels of MDA (Fig. 6B), and decreased levels of GSH (Fig. 6C). Additionally, the activity of GPX4 was reduced in the Hyperoxia + Ad-TXNIP group, as shown in Fig. 6D. Western blot analysis revealed that the protein expression levels of SLC7A11 and GPX4 were significantly decreased in the Hyperoxia + Ad-TXNIP group compared with the Air, Hyperoxia, and Hyperoxia + Ad-vector groups (Fig. 6E). These results indicate that overexpression of TXNIP promotes ferroptosis in BPD.

### Knockdown of TXNIP in vivo alleviates the progression of BPD

**Fig. 7:**
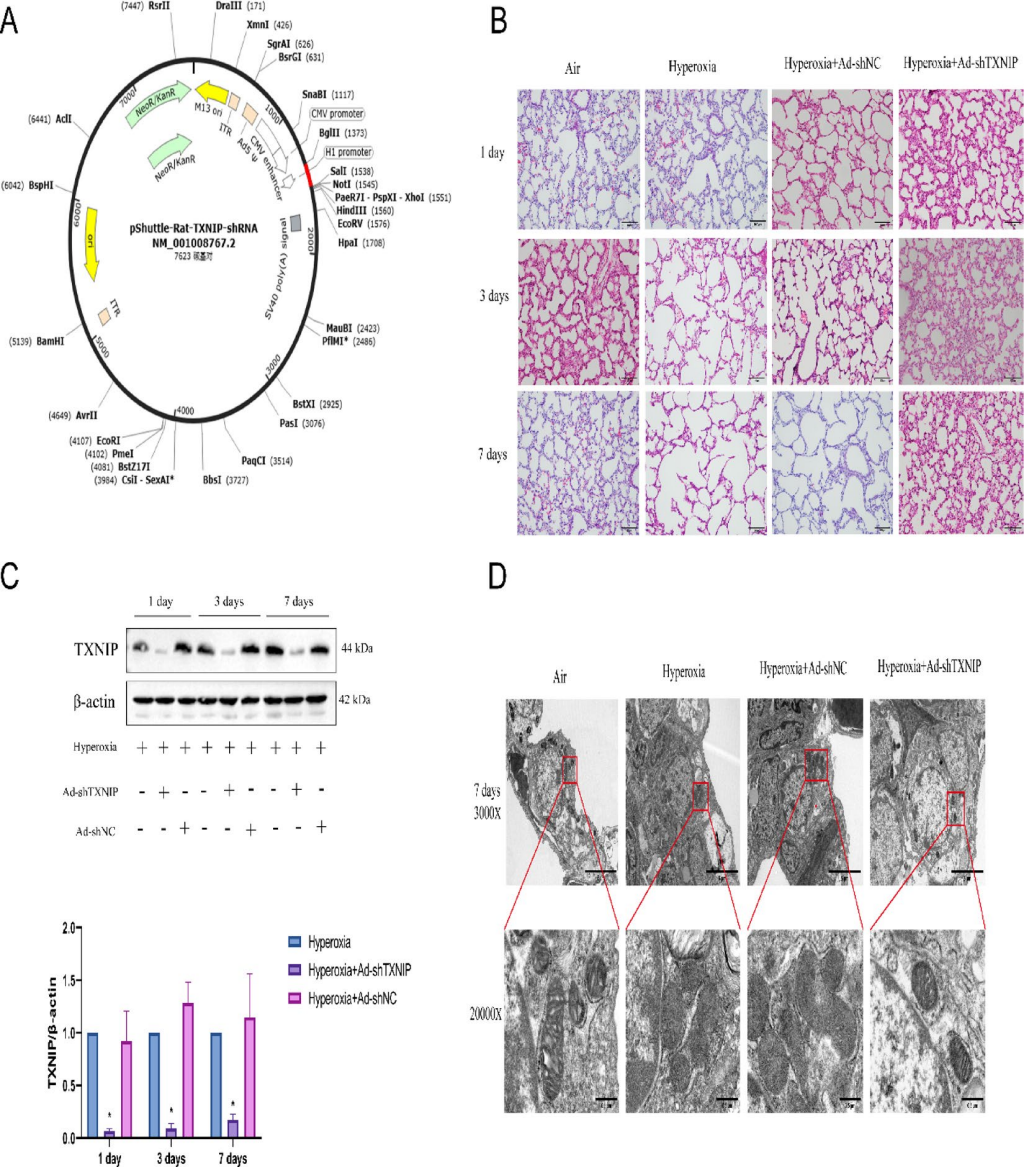
The condition of lung tissues after TXNIP knockout. (**A**) Schematic diagram of the lentiviral vector for TXNIP knockdown. (**B**) H&E staining of lung tissues to assess pathological changes associated with lung injury in the Air, Hyperoxia, Hyperoxia + Ad-shNC and Hyperoxia + Ad-shTXNIP groups on postnatal days 1, 3, and 7. (**C**) Western blot analysis of TXNIP protein expression in lung tissues in the Hyperoxia, Hyperoxia + Ad-shNC and Hyperoxia + Ad-shTXNIP groups on postnatal days 1, 3, and 7 (original WB images are presented in Supplementary information). (**D**) TEM assessment of ultrastructural changes in lung tissue in the Air, Hyperoxia, Hyperoxia + Ad-shNC and Hyperoxia + Ad-shTXNIP groups on postnatal day 7. In the adenovirus rat group, Adenovirus serotype 5 (Ad5) vectors for knockdown of TXNIP were constructed through in vitro transcription. On postnatal day 1, rats in the overexpression groups were anesthetized, and a cervical incision was made to expose the trachea. A 30-gauge needle was used to inject 100 μL of the corresponding adenovirus into the trachea , with an adenovirus titer of 1.8 × 10^8^ pfu. Data are presented as mean ± SD (n = 6); two-way analysis of variance (ANOVA) was used, with Tukey’s post hoc test for inter-group multiple comparisons; * P < 0.05 vs. Hyperoxia.

To investigate the impact of TXNIP downregulation on BPD, we constructed an adenoviral vector to achieve TXNIP knockout in rats. The structure of the TXNIP-knockout adenovirus is shown in Fig. 7A. After in vivo transcription and expression for 1, 3, or 7 days, lung tissues were collected from the rats for western blot analysis. As shown in Fig. 7B, the expression of TXNIP protein in the Hyperoxia + Ad-shTXNIP group was significantly lower than that in the Hyperoxia or Hyperoxia + Ad-shNC groups, indicating successful knockout of TXNIP in the rats. Subsequently, H&E staining was performed. As shown in Fig. 7C, the lung tissues of Air-exposed neonatal rats exhibited uniform alveolar structures with clear cytoplasm and nuclei, and evenly-distributed cells. Compared with the Air group, the Hyperoxia and Hyperoxia + Ad-shNC groups showed thinning of alveolar walls, uneven alveolar sizes, fusion of some alveolar spaces, a significant reduction in alveolar numbers, and simplification of alveolar structures. Additionally, some pulmonary septa were markedly thickened, with evidence of vascular dilation, interstitial proliferation, and fibrosis. Compared with the Hyperoxia and Hyperoxia + Ad-shNC groups, TXNIP knockout significantly improved the pathological changes in lung tissues induced by hyperoxia, as evidenced by increased alveolar numbers and reduced alveolar septal thickness in the Hyperoxia + Ad-shTXNIP group. TEM was also conducted. As shown in Fig. 7D, the mitochondria in the lung tissues of the Air group were intact in shape, with a double-membrane structure and clearly visible cristae. In contrast, the mitochondria in the Hyperoxia and Hyperoxia + Ad-shNC groups exhibited severe structural damage, including mitochondrial shrinkage, vacuolization, and loss of cristae. Compared with the Hyperoxia group, TXNIP knockout significantly alleviated mitochondrial damage, with some mitochondria retaining a double-membrane structure and cristae. These results indicate that TXNIP knockout can mitigate the progression of BPD.

### Knockdown of TXNIP in vivo inhibits ferroptosis in BPD

**Fig. 8:**
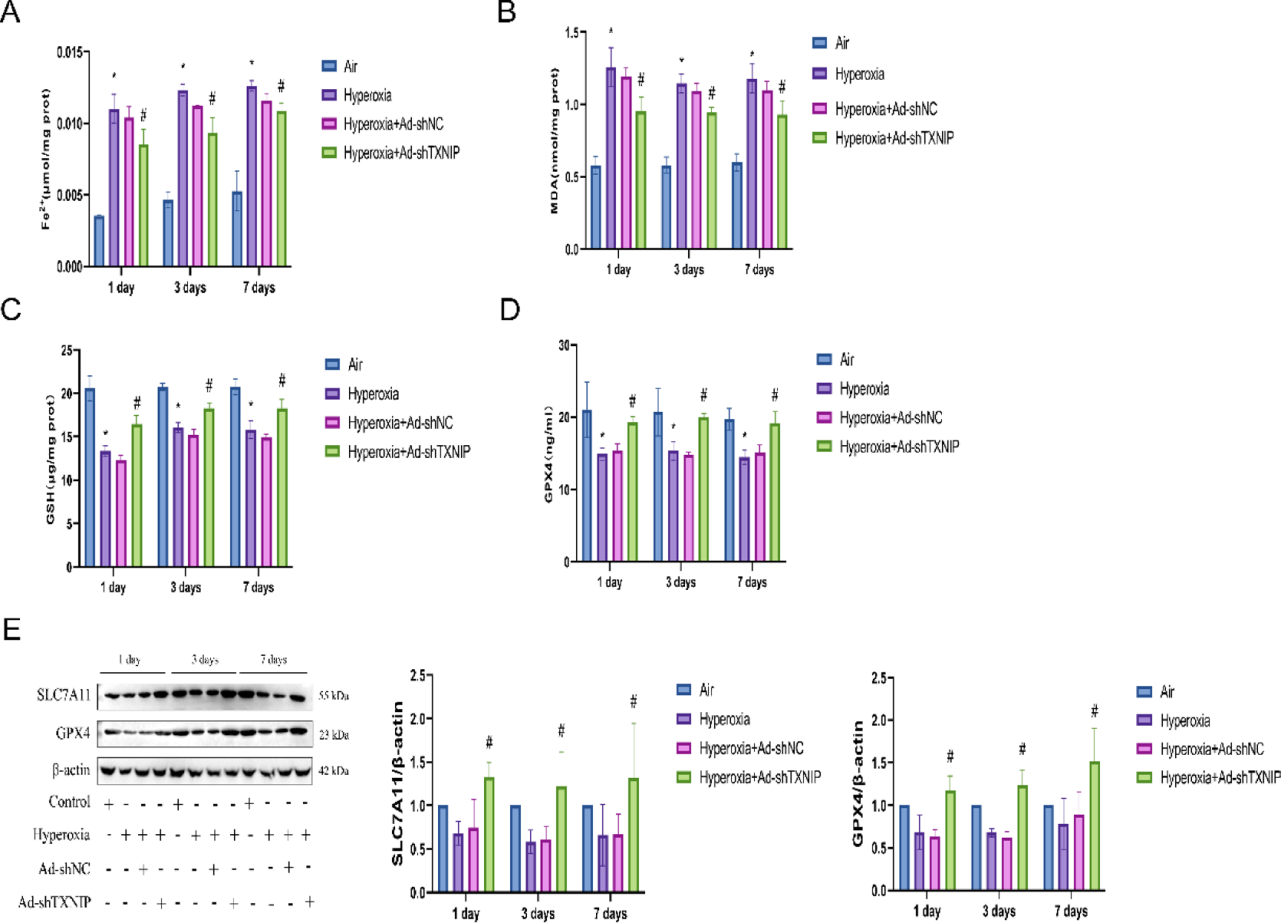
Ferroptosis status following TXNIP knockdown in the BPD model. (**A**–**D**) Quantitative analysis of ferroptosis-related markers in lung tissues from the Air, Hyperoxia, Hyperoxia + Ad-shNC, and Hyperoxia + Ad-shTXNIP groups on postnatal days 1, 3, and 7. (**A**) Measurement of Fe^2+^ levels using an iron assay kit. (**B**) Measurement of MDA levels using an MDA assay kit. C: Measurement of GSH levels using a GSH assay kit. (**D**) Measurement of GPX4 activity using a GPX4 assay kit. (**E**) Western blot analysis of SLC7A11 and GPX4 protein expression in lung tissue in the Air, Hyperoxia, Hyperoxia + Ad-shNC and Hyperoxia + Ad-shTXNIP groups on postnatal days 1, 3, and 7 (original WB images are presented in Supplementary information). In the adenovirus rat group, Adenovirus serotype 5 (Ad5) vectors for knockdown of TXNIP were constructed through in vitro transcription. On postnatal day 1, rats in the overexpression groups were anesthetized, and a cervical incision was made to expose the trachea. A 30-gauge needle was used to inject 100 μL of the corresponding adenovirus into the trachea, with an adenovirus titer of 1.8 × 10^8^ pfu. Data are presented as mean ± SD (n = 6); two-way analysis of variance (ANOVA) followed by Tukey’s post hoc test was used for inter-group multiple comparisons; * *P* < 0.05vs. Air; #* P* < 0.05 vs. Hyperoxia.

To further explore the relationship between TXNIP and ferroptosis in BPD, we knocked down TXNIP in rats and examined the biomarkers of ferroptosis. As shown in Fig. 8A, compared with the Air, Hyperoxia, and Hyperoxia + Ad-shNC groups, the Hyperoxia + Ad-shTXNIP group exhibited significantly reduced levels of ferrous iron (Fe^2+^). Similarly, the MDA levels were decreased in the Hyperoxia + Ad-shTXNIP group (Fig. 8B), while the GSH levels were increased (Fig. 8C), and the activity of GPX4 was elevated (Fig. 8D). Western blot analysis revealed that the protein expression levels of SLC7A11 and GPX4 were significantly increased in the Hyperoxia + Ad-shTXNIP group compared with the Air, Hyperoxia, and Hyperoxia + Ad-shNC groups (Fig. 8E). These results indicate that knockdown of TXNIP inhibits ferroptosis in BPD.

### TXNIP downregulates the SLC7A11/GPX4 pathway to promote ferroptosis in BPD

**Fig. 9:**
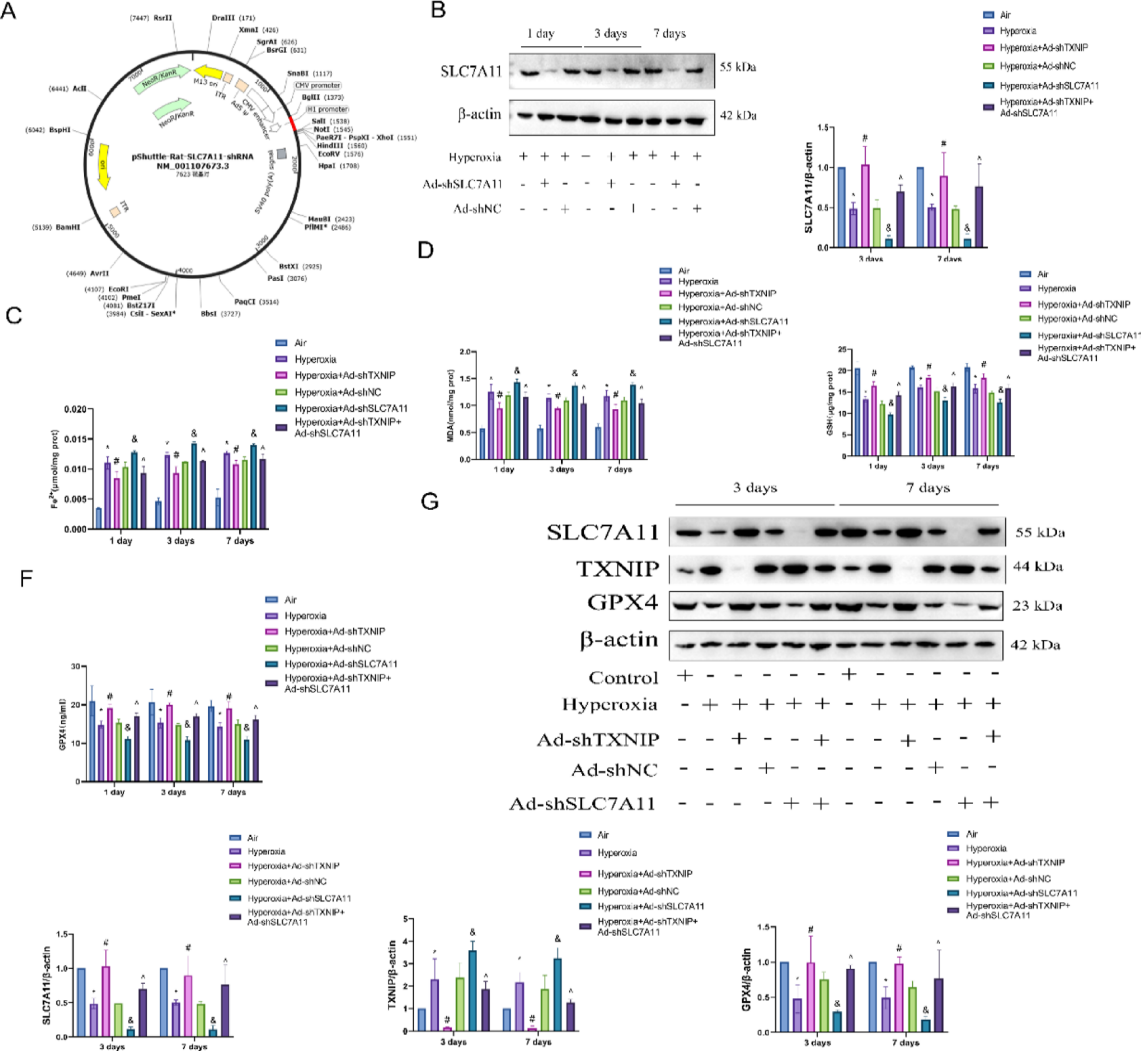
Mechanism via which TXNIP regulates ferroptosis in BPD. (**A**) Schematic diagram of the lentivirus structure for SLC7A11 knockdown. (**B**) Western blot analysis of SLC7A11 protein expression in lung tissue in the Hyperoxia, Hyperoxia + Ad-shNC and Hyperoxia + Ad-shSLC7A11 groups on postnatal days 1, 3, and 7 (original WB images are presented in Supplementary information). (**C**–**F**) Quantitative assessments of ferroptosis-related biochemical markers across the indicated groups (Air, Hyperoxia, Hyperoxia + Ad-shNC, Hyperoxia + Ad-shTXNIP, Hyperoxia + Ad-shSLC7A11, and Hyperoxia + Ad-shTXNIP + Ad-shSLC7A11) on postnatal days 1, 3, and 7. C: Measurement of Fe^2+^ levels using an iron assay kit. (**D**) Measurement of MDA levels using an MDA assay kit. (**E**) Measurement of GSH levels using a GSH assay kit. (**F**) Measurement of GPX4 activity using a GPX4 assay kit. G: Western blot analysis of SLC7A11, TXNIP, and GPX4 protein expression in lung tissue in the Air, Hyperoxia, Hyperoxia + Ad-shNC, Hyperoxia + Ad-shTXNIP, Ad-shSLC7A11 and Hyperoxia + Ad-shTXNIP + Ad-shSLC7A11 groups on postnatal days 3 and 7. (original WB images are presented in Supplementary information). In the adenovirus rat group, Adenovirus serotype 5 (Ad5) vectors for knockdown of TXNIP and SLC7A11 were constructed through in vitro transcription. On postnatal day 1, rats in the overexpression groups were anesthetized, and a cervical incision was made to expose the trachea. A 30-gauge needle was used to inject 100 μL of the corresponding adenovirus into the trachea, with an adenovirus titer of 1.8 × 10^8^ pfu. Data are presented as mean ± SD (n = 6); two-way analysis of variance (ANOVA) combined with Tukey’s post hoc test was used for inter-group multiple comparisons; * P < 0.05 vs. Air; #* P* < 0.05 vs. Hyperoxia; &* P* < 0.05 vs. Hyperoxia + Ad-shNC; ^ *P* < 0.05 vs. Hyperoxia + Ad-shSLC7A11.

To investigate the mechanisms underlying the role of TXNIP in ferroptosis in BPD, we constructed an adenoviral vector to knock down the upstream factor, SLC7A11, in the signaling pathway. The structure of the adenovirus targeting SLC7A11 is shown in Fig. 9A. After transduction and expression for 1, 3, or 7 days in vivo, lung tissues were collected from rats for western blot analysis. As shown in Fig. 9B, the protein expression of SLC7A11 was significantly decreased in the Hyperoxia + Ad-shSLC7A11 group compared with the Hyperoxia and Hyperoxia + Ad-shNC groups, indicating successful knockdown of SLC7A11 in the rats. We then examined the biomarkers of ferroptosis. Compared with the Hyperoxia group, the levels of ferrous iron (Fe^2+^) were decreased in the Hyperoxia + Ad-shTXNIP group, but increased in the Hyperoxia + Ad-shSLC7A11 group. However, this increase was reversed in the Hyperoxia + Ad-shTXNIP + Ad-shSLC7A11 group (Fig. 9C). Similarly, the levels of MDA were decreased in the Hyperoxia + Ad-shTXNIP group, but increased in the Hyperoxia + Ad-shSLC7A11 group, and this increase was reversed in the Hyperoxia + Ad-shTXNIP + Ad-shSLC7A11 group (Fig. 9D). In contrast, the levels of GSH were increased in the Hyperoxia + Ad-shTXNIP group, but decreased in the Hyperoxia + Ad-shSLC7A11 group, and this decrease was reversed in the Hyperoxia + Ad-shTXNIP + Ad-shSLC7A11 group (Fig. 9E). Additionally, the activity of GPX4 was increased in the Hyperoxia + Ad-shTXNIP group, but decreased in the Hyperoxia + Ad-shSLC7A11 group, and this decrease was reversed in the Hyperoxia + Ad-shTXNIP + Ad-shSLC7A11 group (Fig. 9F). Western blot analysis further revealed that, compared with the Hyperoxia group, the protein expression of TXNIP was decreased, while the expression of both SLC7A11 and GPX4 was increased in the Hyperoxia + Ad-shTXNIP group. Conversely, the protein expression of TXNIP was increased, while the expression of SLC7A11 and GPX4 was decreased in the Hyperoxia + Ad-shSLC7A11 group. These changes were reversed in the Hyperoxia + Ad-shTXNIP + Ad-shSLC7A11 group (Fig. 9G). These results indicate that TXNIP downregulates the SLC7A11/GPX4 pathway to promote ferroptosis in BPD.

## Discussion

Bronchopulmonary dysplasia (BPD) is characterized by arrested lung development and abnormal pulmonary microvascular development, presenting as reduced alveolar numbers, enlarged alveolar volume, simplified alveolar structure, and abnormal morphology of pulmonary microvessels, with relatively mild alveolar and airway injury and fibrosis^1,27^. Currently, clinical treatments for BPD are primarily supportive and symptomatic, including management of respiration and circulation, nutritional support, and the use of drugs such as corticosteroids, diuretics, and bronchodilators, with no definitive and effective preventive or therapeutic measures^28–30^.

Thioredoxin-interacting protein (TXNIP) was first discovered in 1994^16^. TXNIP plays a crucial regulatory role in the pathophysiological processes of various diseases, such as triggering inflammatory responses, enhancing oxidative stress, and mediating apoptosis^31,32^. Previous studies have shown that TXNIP is involved in the development of BPD^24,25^. In this study, we used a BPD animal model to simulate clinical BPD in infants. After modeling, we analyzed the expression levels of TXNIP in lung tissues using RT-qPCR and western blotting, and observed pulmonary morphological changes after knocking down or overexpressing TXNIP to verify its involvement in BPD development. The results showed that, compared with the Air control group, the mRNA and protein expression levels of TXNIP were elevated in the BPD model group. H&E staining and TEM examination revealed that overexpression of TXNIP exacerbated lung injury, while knockdown of TXNIP significantly improved lung injury. These findings indicate that increased TXNIP expression in BPD exacerbates the condition, consistent with previous studies.

Programmed cell death (PCD), including apoptosis, necrosis, autophagy, pyroptosis, and ferroptosis, plays a significant role in the molecular and biological mechanisms and further progression of BPD^33^. This study confirmed that ferroptosis is involved in the development of BPD. Ferroptosis, a novel form of PCD, was first proposed by Dixon et al. in 2012^8^. Biochemically, ferroptosis is characterized by Fe^2+^ overload, depletion of GSH and GPX4, and excessive accumulation of lipid reactive oxygen species (ROS)^8^ GPX4 is a key enzyme in the ferroptosis pathway^34^. Generally, levels of GPX4, MDA, 4-hydroxynonenal (4-HNE), and SLC7A11 are considered indicators of ferroptosis severity. In this study, we found severe mitochondrial damage in BPD model samples, characterized by shrinkage, vacuolization, and loss of cristae. Compared with the Air control group, the BPD model group exhibited increased levels of MDA and Fe^2+^, decreased GSH levels, and reduced GPX4 activity. These data indicate that hyperoxia exposure induces ferroptosis in pulmonary epithelial cells of neonatal SD rats, consistent with previous studies^9–12^. We also treated neonatal rats in the BPD model group with ferroptosis inducers and inhibitors, and assessed lung injury pathology via H&E staining, observed ultrastructural changes in lung tissue cells using TEM, and measured ferroptosis biomarkers (Fe^2+^, MDA, GSH levels, and GPX4 activity) and protein expression of SLC7A11 and GPX4 using western blotting. The results showed that modulating ferroptosis regulates hyperoxic lung injury, with promotion of ferroptosis exacerbating BPD.

**Fig. 10:**
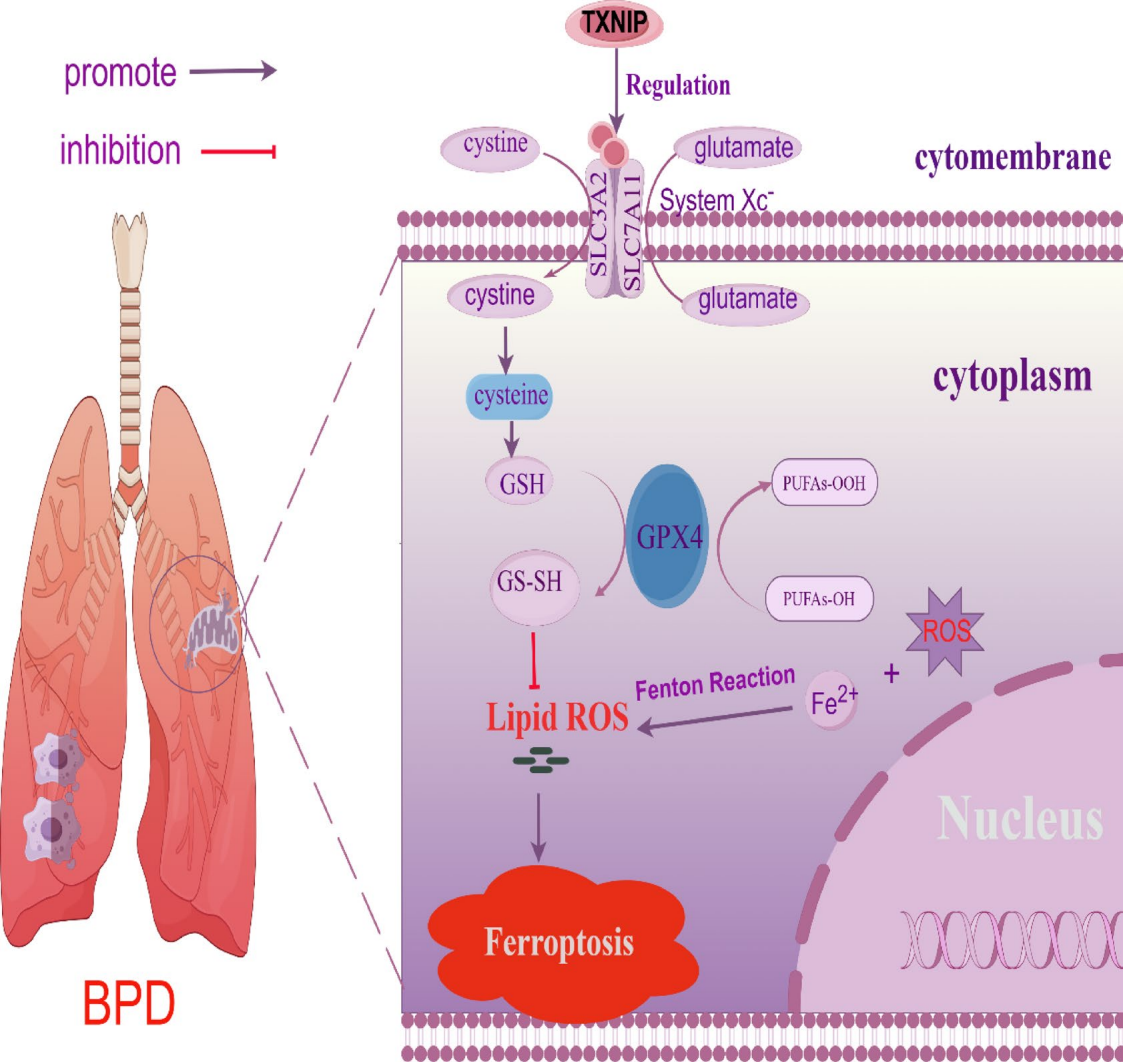
Mechanism via which TXNIP regulates SLC7A11/GPX4 pathway-mediated ferroptosis in BPD. TXNIP, thioredoxin-interacting protein; SLC7A11, solute carrier family 7 member 11; SLC3A2, solute carrier family 3, member 2**;** GPX4, glutathione peroxidase 4; GSH, glutathione; GS-SH, glutathione-S-sulfhydrate; PUFAs-OH, polyunsaturated fatty acids-OH; PUFAs-OOH, polyunsaturated fatty acids-oxygenated species; lipid ROS, lipid reactive oxygen species; ROS, reactive oxygen species; Fe^2+^, ferrous Iron; BPD, bronchopulmonary dysplasia.

The pathogenesis of BPD is complex and multifactorial, involving both genetic and environmental factors. The underlying mechanisms are associated with various prenatal, perinatal, and postnatal factors. Prenatal factors include intrauterine growth restriction, maternal diseases, and infections. Perinatal factors encompass prematurity, low birth weight, and gender, while postnatal factors involve hyperoxia, ventilator-induced barotrauma, and infections^33^. Research has shown that oxidative stress, resulting from an imbalance between oxidative and antioxidant systems, is the primary form of damage in BPD^35^. Under physiological conditions, TXNIP is localized in the nucleus^36^. Under excessive ROS conditions, TXNIP expression is upregulated due to the inhibition of Adenosine 5′-monophosphate-activated protein kinase (AMPK) phosphorylation and the translocation of TXNIP from the nucleus to the cytoplasm, leading to endoplasmic reticulum and mitochondrial stress^37^. The translocation of TXNIP between the nucleus and cytoplasm promotes its interaction with thioredoxin-1 (Trx1), resulting in inhibition of Trx1 activity and the subsequent onset of various diseases. TXNIP binds to thioredoxin (Trx), preventing its normal antioxidant function, promoting ROS accumulation, and causing oxidant/antioxidant imbalance. This triggers endoplasmic reticulum and mitochondrial stress, ultimately leading to inflammation or apoptosis^32^. Studies have shown that TXNIP promotes ferroptosis^38^. Moreover, it has been found that TXNIP actually initiates ferroptosis and eventually contributes to high fructose-induced renal tubular epithelial cell damage^39^. We hypothesized that TXNIP regulates ferroptosis in BPD. In this study, we used adenovirus serotype 5 (Ad5) as a vector to overexpress or knock down TXNIP, then evaluated levels of ferroptosis-related markers and measured the expression levels of SLC7A11 and GPX4 using western blotting. The results showed that overexpression of TXNIP promotes ferroptosis in BPD, while knockdown of TXNIP inhibits it, consistent with our hypothesis. Previous studies have shown that modulating the SLC7A11/GPX4 pathway regulates ferroptosis^14,15^. Furthermore, studies have suggested that TXNIP expression may be correlated with the expression of GPX4 and SLC7A11^26^. Therefore, to further explore whether TXNIP regulates ferroptosis in BPD via the SLC7A11/GPX4 signaling pathway, we knocked down the upstream factor SLC7A11 in the pathway, then analyzed ferroptosis-related markers and measured the expression levels of TXNIP, SLC7A11, and GPX4 using western blotting. Our results indicate that regulation of ferroptosis by TXNIP is dependent on the SLC7A11/GPX4 signaling pathway (Fig. 10).

In summary, TXNIP downregulates the SLC7A11/GPX4 pathway to promote ferroptosis in BPD model mice. These findings suggest that gene therapy targeting TXNIP using adenovirus as a vector is a potential therapeutic strategy for BPD, providing new insights and targets for the clinical treatment of BPD. However, one limitation of this study is the lack of validation using human preterm lung tissues. Although our findings in the neonatal rat model provide important mechanistic insights into the role of TXNIP and ferroptosis in BPD, clinical validation is currently lacking due to the limited availability of well-preserved and ethically approved human specimens. Validation of these findings in human samples would further enhance their translational relevance. Therefore, in future studies, we plan to incorporate clinical samples to further validate our findings and assess their translational relevance. The inclusion of patient-derived data will help bridge the gap between experimental animal models and human disease.

## Materials and methods

### Experimental animals—grouping and treatment

Newborn Sprague–Dawley (SD) rats were procured from Liaoning Changsheng Biotechnology Co., Ltd. The rats were randomly divided into ten groups: Air, Hyperoxia, Hyperoxia + RSL3, Hyperoxia + Ferrostatin-1, Hyperoxia + Ad-shNC, Hyperoxia + Ad-shTXNIP, Hyperoxia + Ad-vector, Hyperoxia + Ad-TXNIP, Hyperoxia + Ad-shSLC7A11, and Hyperoxia + Ad-shTXNIP + Ad-shSLC7A11. A hyperoxia-induced BPD animal model was established as previously described^40^. The Air group was maintained in room air with an oxygen concentration of 21%. The Hyperoxia group was placed in a sealed plexiglass hyperoxia chamber with an oxygen concentration of 85%, temperature of 25–26 °C, humidity of 60–70%, and carbon dioxide concentration < 0.5%. To prevent oxygen toxicity in the mother rats, which could affect their nursing ability, the mother rats of the Hyperoxia and Air groups were exchanged every 24 h. The chamber was opened daily for 30 min to replenish bedding, clean water, and food. In the Hyperoxia + Ferrostatin-1 group, rats were intraperitoneally injected with 10 mg/kg of the ferroptosis inhibitor Ferrostatin-1 (Aladdin Chemistry Co. Ltd., Shanghai, China) daily for 3 days^41^. In the Hyperoxia + RSL3 group, rats were intraperitoneally injected with 5 mg/kg of the ferroptosis activator RSL3 (Macklin Biochemical Company Ltd., Shanghai, China) daily for 6 days^42^. Adenovirus serotype 5 (Ad5) vectors for overexpression of TXNIP and knockdown of TXNIP and SLC7A11 were constructed through in vitro transcription (Wanlei Biotechnology, Shenyang, China). On postnatal day 1, rats in the overexpression and knockdown groups were anesthetized using inhalational isoflurane (3% for induction and 1.5% for maintenance), and a cervical incision was made to expose the trachea^43^. A 30-gauge needle was used to inject 100 μL of the corresponding adenovirus into the trachea^44^, with an adenovirus titer of 1.8 × 10^8^ pfu^44^. Lung tissues were collected from each group on postnatal days 1, 3, and 7 for subsequent experiments. The study’s overall design and protocol were approved by the Laboratory Animal Ethics Committee of Hainan Medical University (HYLL-2021–390). The animal experiments conducted in this study were performed in accordance with the ARRIVE guidelines, and all methods were performed in accordance with the relevant guidelines and regulations.

### Hematoxylin and eosin (H&E) staining

Lung tissues were fixed in 4% paraformaldehyde, embedded in paraffin, and cut into 5 μm sections. The paraffin sections were baked in a 60 °C oven for 30 min to enhance adhesion, followed by deparaffinization and rehydration. The sections were then stained with hematoxylin to visualize cell nuclei and counterstained with eosin to highlight cytoplasmic and extracellular matrix components. After staining, the sections were dehydrated, cleared, and mounted with neutral resin. Finally, the stained sections were observed under a low-power microscope (Olympus BX53 Microscope, Olympus Corporation, Japan) to assess histological changes. Moreover, Adobe Photoshop 2024 was used to change the brightness and contrast of the image; this was applied equally across the entire image and was applied equally to controls.

### Transmission electron microscopy (TEM)

TEM was used to observe ultrastructural changes in the lung tissue cells. The tissues were first fixed in 2% glutaraldehyde solution at 4 °C, followed by gradient dehydration through 50–90% ethanol solutions. The tissues were then embedded in acetone and Epon 812 at room temperature for 4 h, and ultrathin sections were prepared. Double staining with uranyl acetate and citrate was performed at room temperature for 30 min, and the ultrastructure of the mitochondrial cells in each group was observed using a transmission electron microscope (HT7800, Hitachi, Japan) at 80.0 kV.

### Reverse transcription quantitative polymerase chain reaction (RT-qPCR)

Total RNA was extracted using an RNA purification kit (Takara Bio Inc., Shiga, Japan). cDNA was synthesized using a reverse transcription kit (Takara). Quantitative PCR was performed using a fluorescent quantitative PCR kit (Takara). The primer sequences for the target genes are provided below. Relative expression levels were quantified using the 2^−△△Ct^ method, with GAPDH as the internal reference gene. Table 1 lists the primer sequences.

**Table 1:** Specific primer sequences for RT-qPCR.

Gene	Forward (5′ → 3′)	Reverse (5′ → 3′)
GAPDH	AACGACCCCTTCATTGACCTC	CGCCAGTAGACTCCACGACATA
TXNIP	TACAGGTGAGAACGAGATGGTGA	TTGAGTTGGCTGGCTGGGAC
SLC7A11	GCTGACACTCGTGCTATT	ATTCTGGAGGTCTTTGGT
GPX4	CATCGACGGGCACATGGTCT	CCACACTCAGCATATCGGGCAT
TGF‐β	CCCGAAGCGGACTACTATGC	GCTTCCCGAATGTCTGACGTA
VEGF‐α	GCCAGCACATAGGAGAGATGAG	GGCTTTGTTCTGTCTTTCTTTGG

### Western blot (WB) analysis of TXNIP, SLC7A11, and GPX4 protein expression

Lung tissues from animals in each experimental group were lysed using RIPA lysis buffer (Servicebio, Wuhan, China) to extract total protein. The protein concentration was quantified using a BCA protein assay kit (Solarbio, Beijing, China). Equal amounts of total protein were separated by sodium dodecylsulfate polyacrylamide gel electrophoresis (SDS-PAGE) and then transferred to a polyvinylidene difluoride (PVDF) membrane using a wet transfer system. The membrane was blocked with 5% non-fat dried milk for 2 h at room temperature to prevent non-specific binding. Subsequently, the membrane was incubated overnight at 4 °C with primary antibodies diluted in blocking buffer: TXNIP (1:500) (Wanlei Biotechnology), SLC7A11 (1:500) (Proteintech, Rosemont, IL, USA), GPX4 (1:500) (Servicebio) and β-actin (1:5000) (Servicebio). After washing the membrane three times with TBST, it was incubated with a horseradish peroxidase (HRP)-conjugated secondary antibody (Proteintech) for 1 h at room temperature. The membrane was washed three times again, and protein bands were visualized using an ECL chemiluminescence reagent (Biosharp, Hefei, China). The chemiluminescent signals (Tanon, Shanghai, China) were captured using a gel imaging system. The band intensity was quantified using ImageJ software (version2.3.0, NIH, Bethesda, MD, USA).

### Enzyme-linked immunosorbent assay (ELISA) for GPX4 activity

GPX4 activity in lung tissues from animals in each group was measured using an ELISA kit (Boyuan Biotechnology, Wuhan, China) according to the manufacturer’s instructions. GPX4 activity was determined by measuring the absorbance of each group using a standard protein curve.

### Evaluation of ferrous iron (Fe^2+^) levels

Ferrous iron levels in the lung tissues from each group were determined using a ferrous iron assay kit (Solarbio) according to the manufacturer’s instructions. The absorbance at 593 nm was measured using a microplate reader (SpectraMax i3x, Molecular Devices, Sunnyvale, CA, USA).

### Detection of malondialdehyde (MDA) levels

MDA levels in lung tissues from each group were measured using an MDA assay kit (Solarbio) according to the manufacturer’s instructions. The absorbance at 532 nm and 600 nm was measured using a microplate reader (SpectraMax i3x,Molecular Devices).

### Detection of GSH levels

GSH levels in lung tissues from each group were measured using a GSH assay kit (Solarbio) following the manufacturer’s instructions. The absorbance at 412 nm was measured using a microplate reader (SpectraMax i3x, Molecular Devices).

### Statistical analysis

All statistical analyses were performed using GraphPad Prism 9 (GraphPad Software Inc., San Diego, CA, USA). Data conforming to a normal distribution are presented as the mean ± standard deviation (SD). Comparisons between multiple groups were made using two-way analysis of variance (ANOVA) combined with Tukey’s post hoc test for inter-group multiple comparisons. In the figures, *, #, & and ^ denote the differences between groups as specified in the figure, and the term ‘vs’ denotes the reference group used for comparison at the same time point. The *P*-values represent the statistical significance levels of the comparisons among the groups*. P* < 0.05 was considered statistically significant. *P* > 0.05 was considered not statistically significant.

## Supplementary Information

Below is the link to the electronic supplementary material.

Supplementary Material 1

## Data Availability

The authors declare that the data supporting the findings of this study are available within the paper and its Supplementary Information files. Should any raw data files be needed in another format they are available from the corresponding author upon reasonable request.
