# Supplementary material for: TXNIP mediates ferroptosis in a bronchopulmonary dysplasia mouse model by regulating the SLC7A11/GPX4 pathway
Source: Sci Rep. 2025 Oct 8;15:35188. doi: 10.1038/s41598-025-19092-6 (PMC12508065; doi:10.1038/s41598-025-19092-6)

Supplementary Information for

TXNIP Mediates Ferroptosis in Bronchopulmonary Dysplasia Mice Model by Regulating the SLC7A11/GPX4 Pathway

Dongzhui Chen, Feifei Yin, Pin Yang, Wanrong Xia, Yi Huang, Yue Feng, Li Yang Shuqiang Lin, Qiuyue Zhang*

* *Corresponding:* hy0203157@muhn.edu.cn.

Fig 2E

β-actin

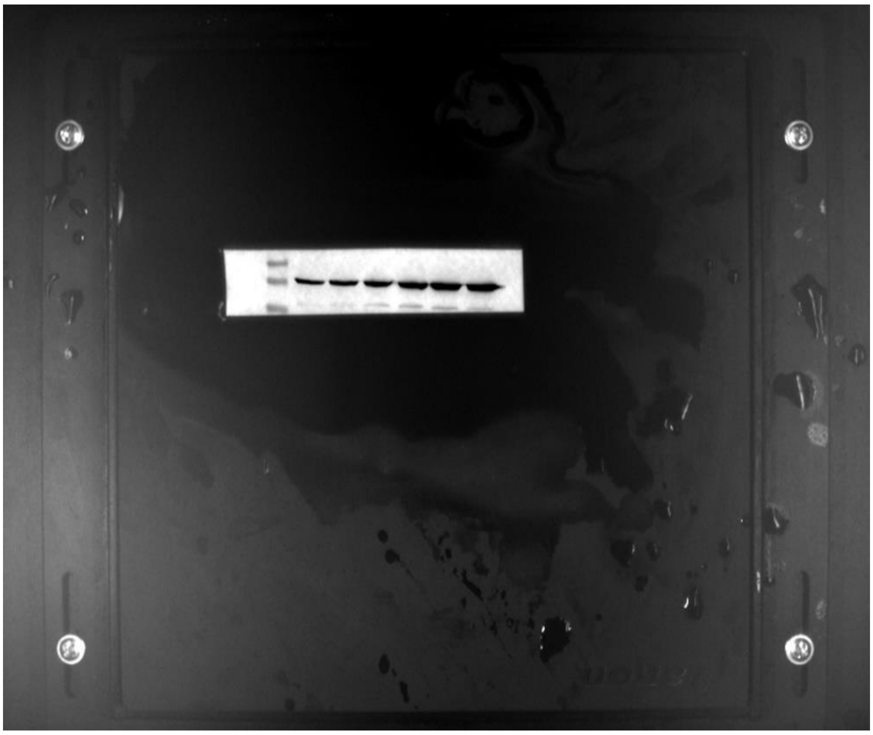

TXNIP

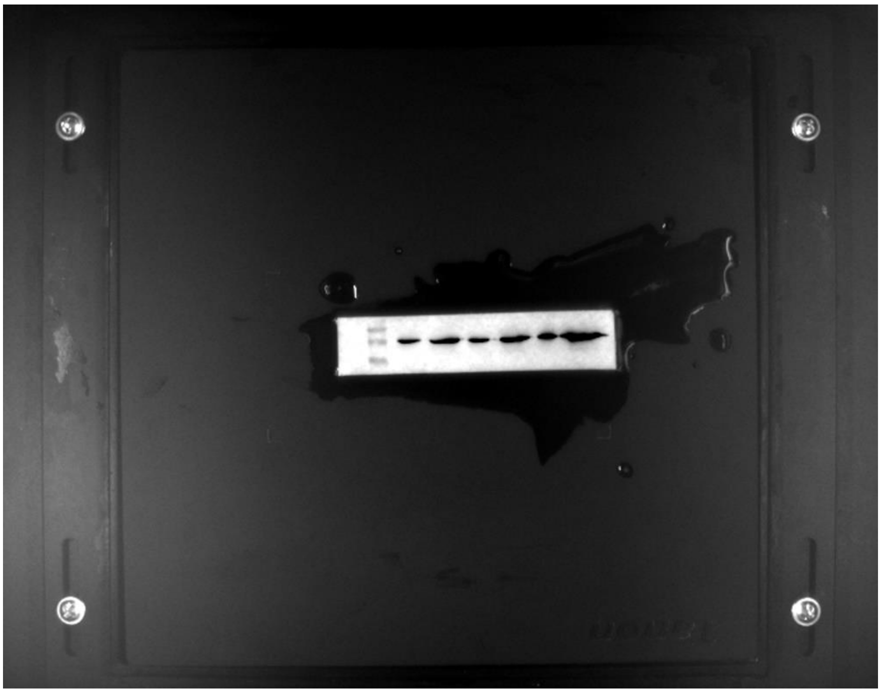

Fig 3G

β -actin

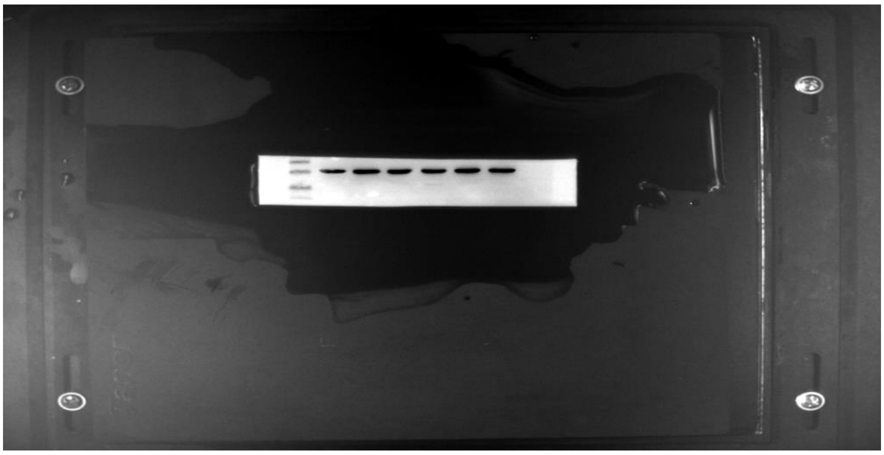

GPX4

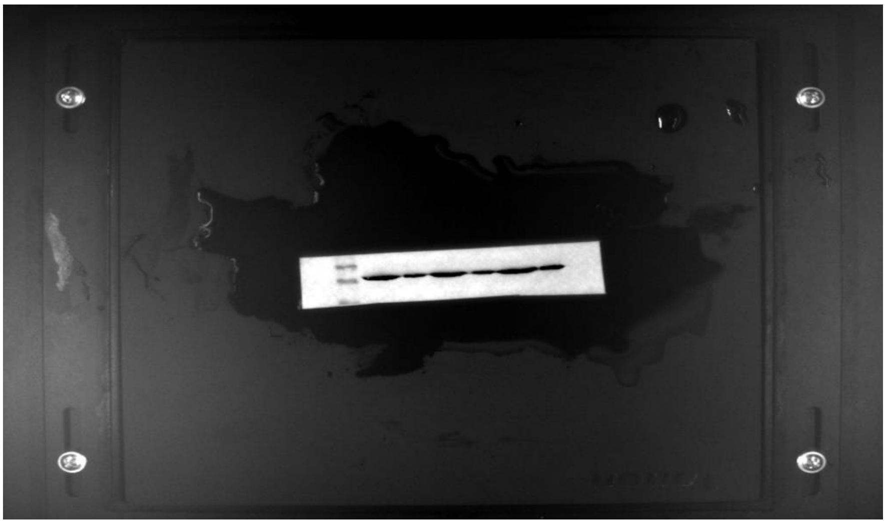

SLC7A11

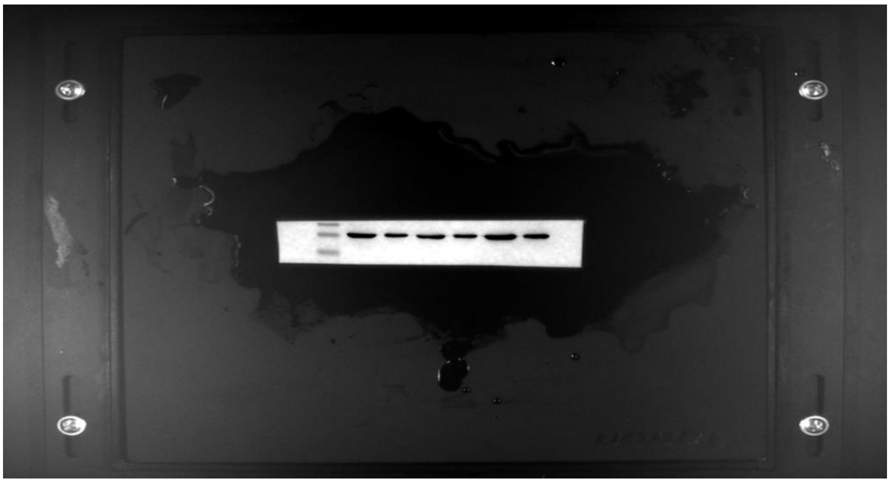

Fig 4G

β -actin

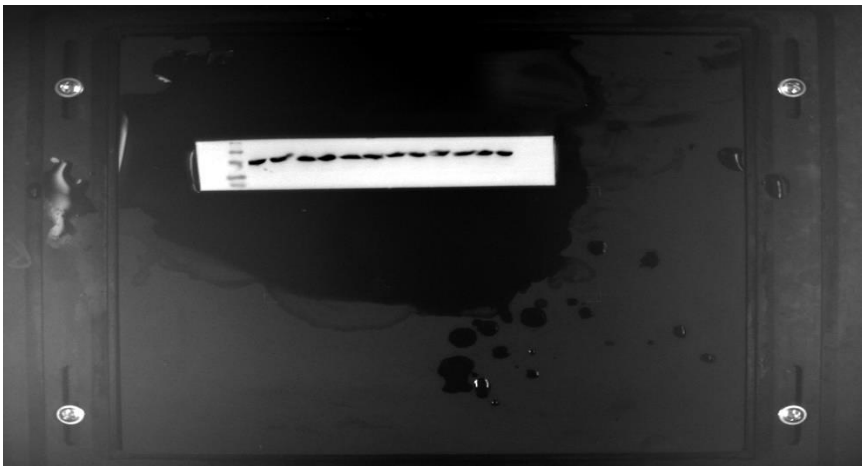

GPX4

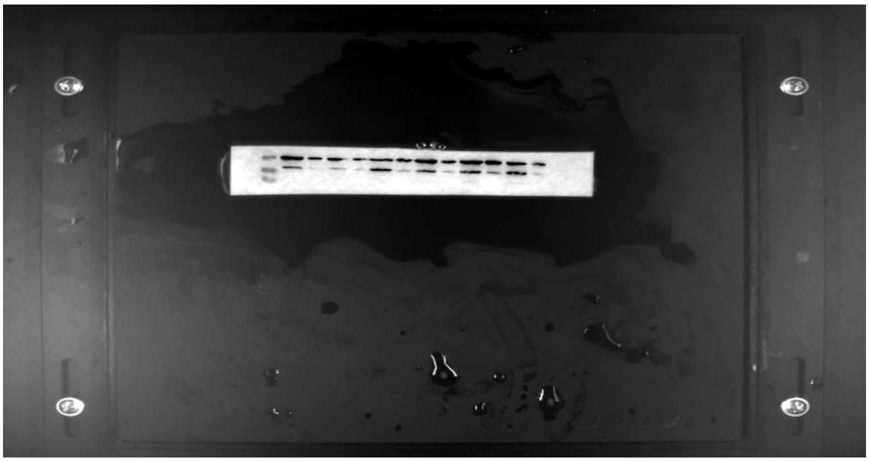

SLC7A11

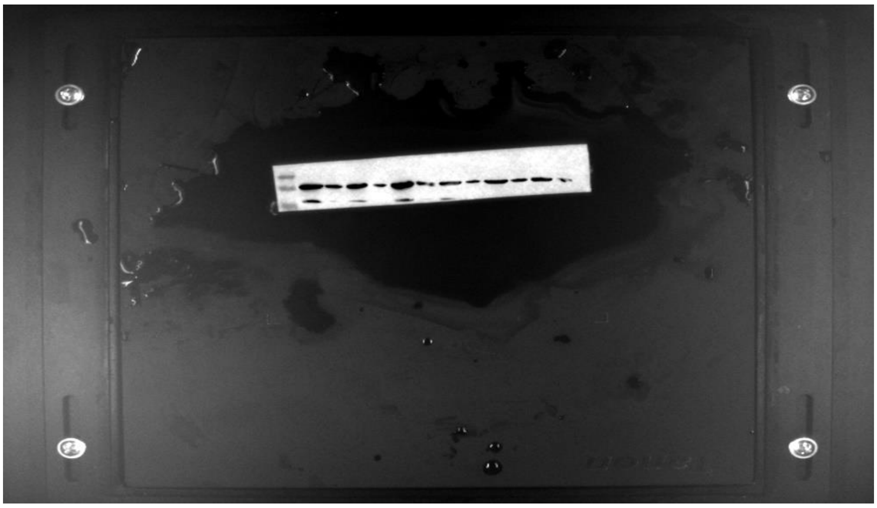

Fig 5B

β -actin

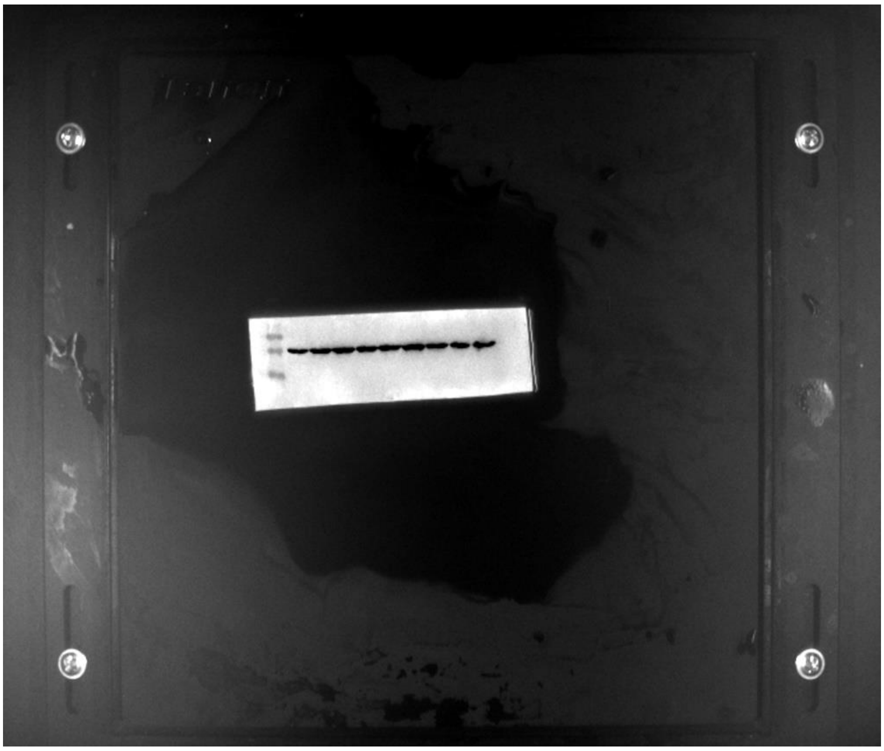

TXNIP

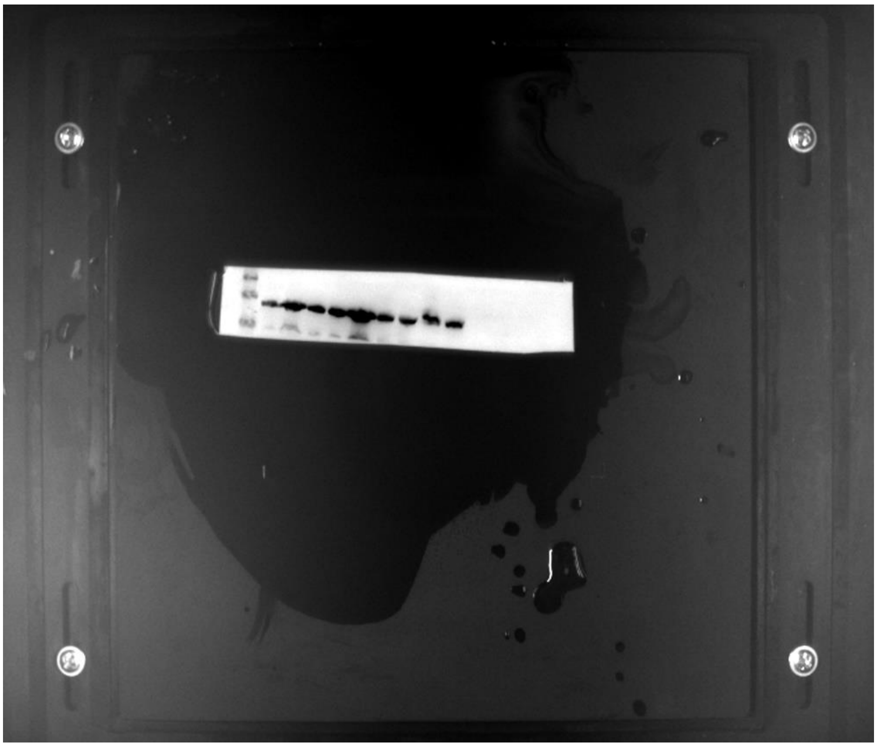

Fig 6E

β -actin

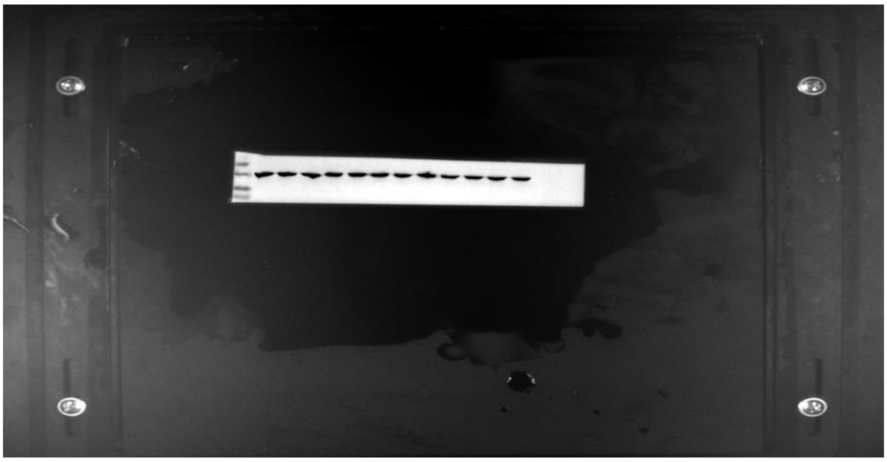

GPX4

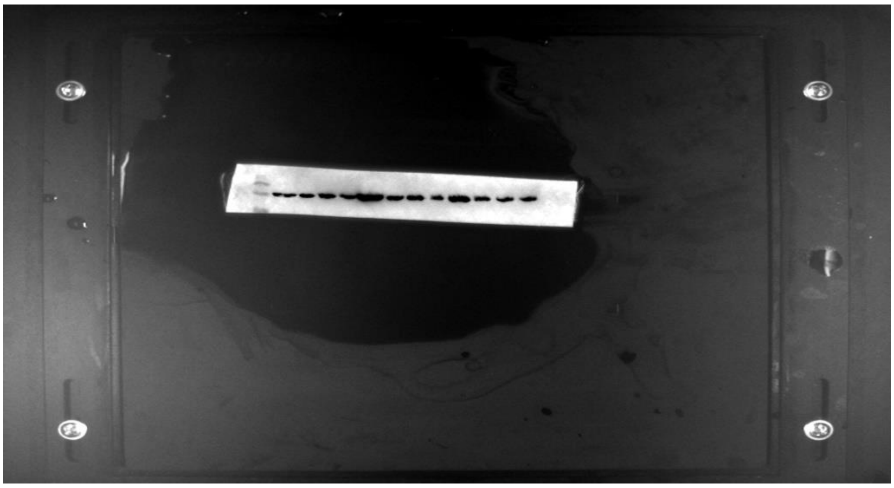

SLC7A11

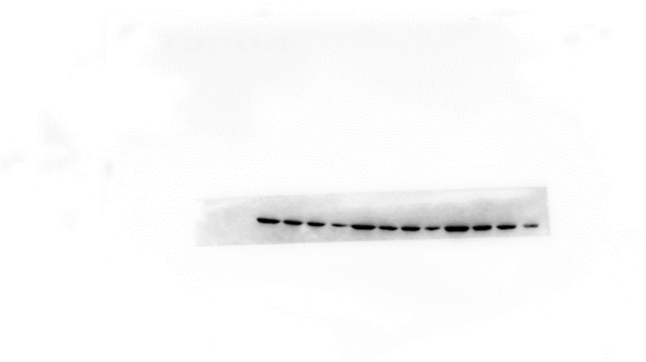

Fig 7C

β -actin

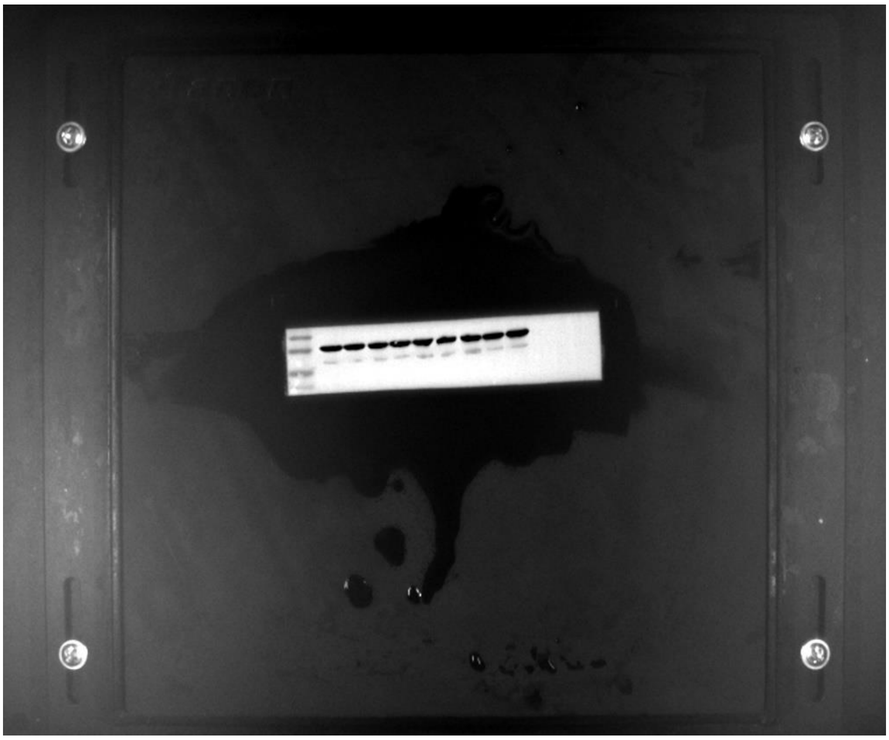

TXNIP

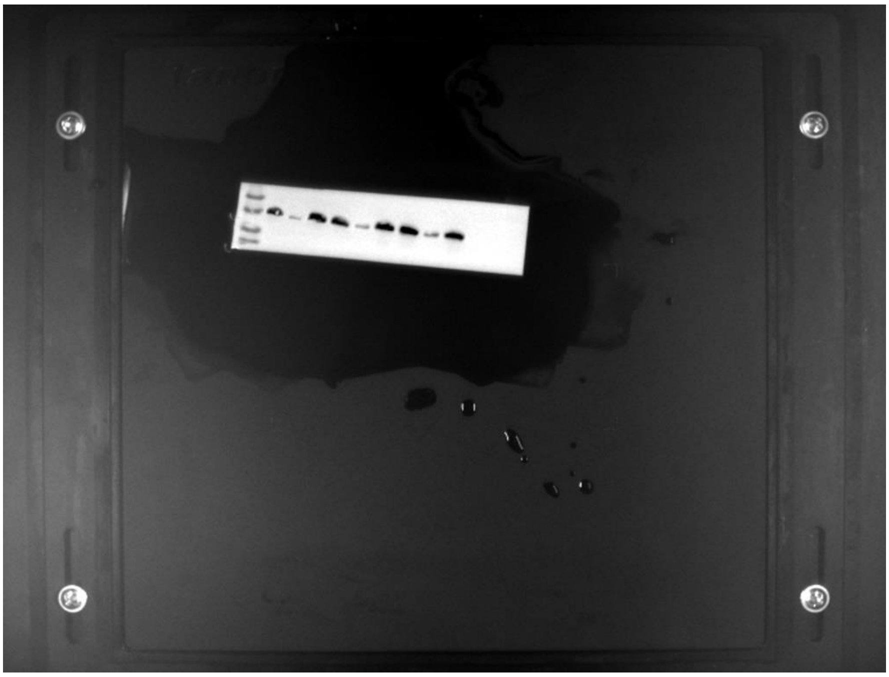

Fig 8E

β -actin

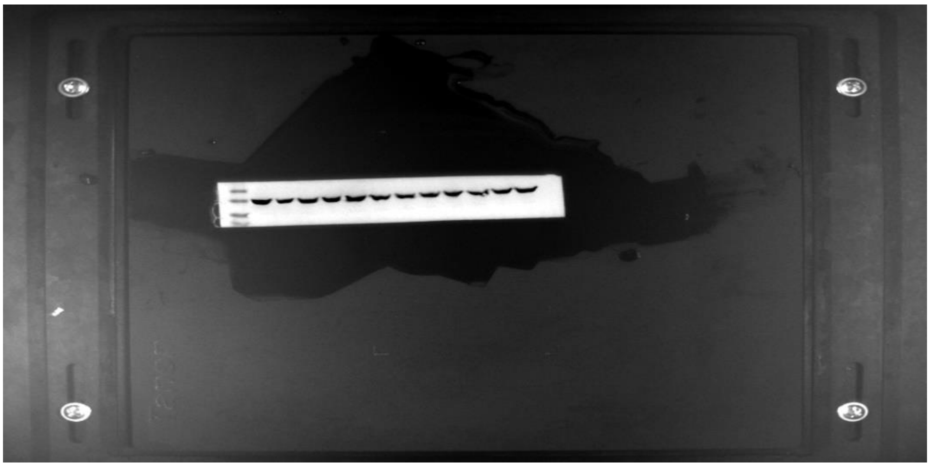

GPX4

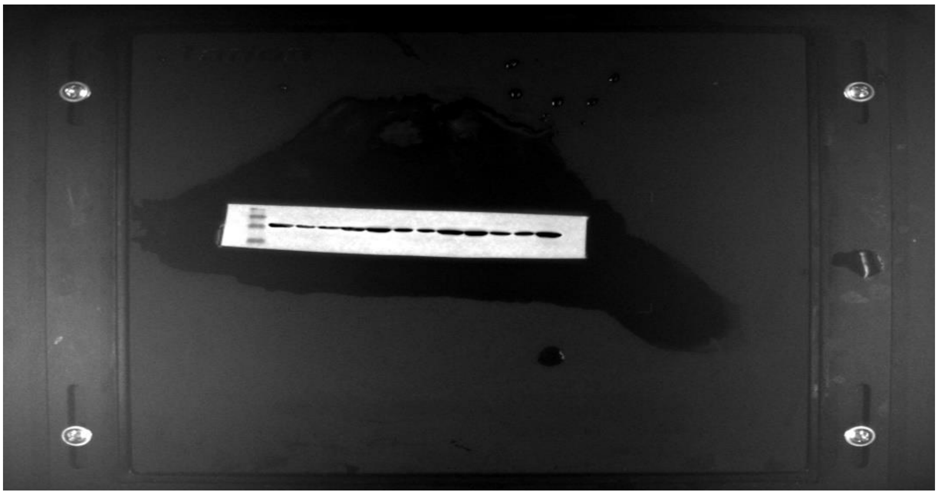

SLC7A11

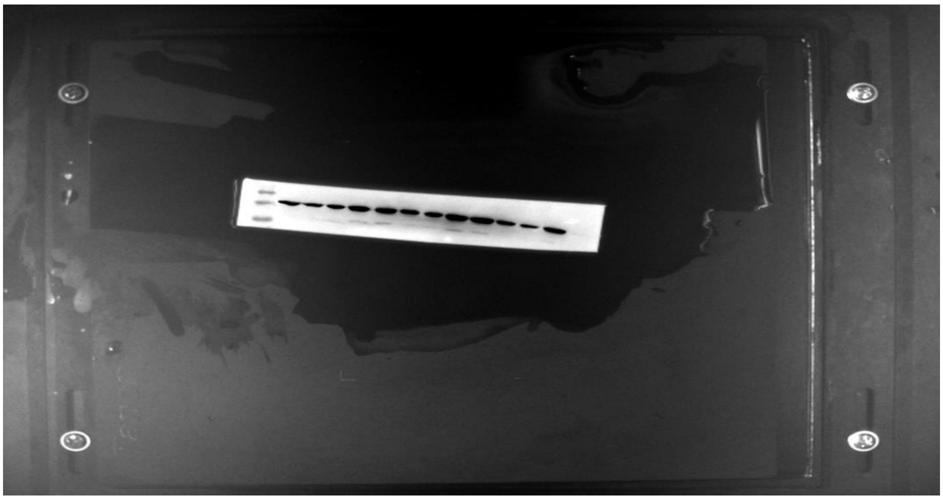

Fig 9B

β -actin

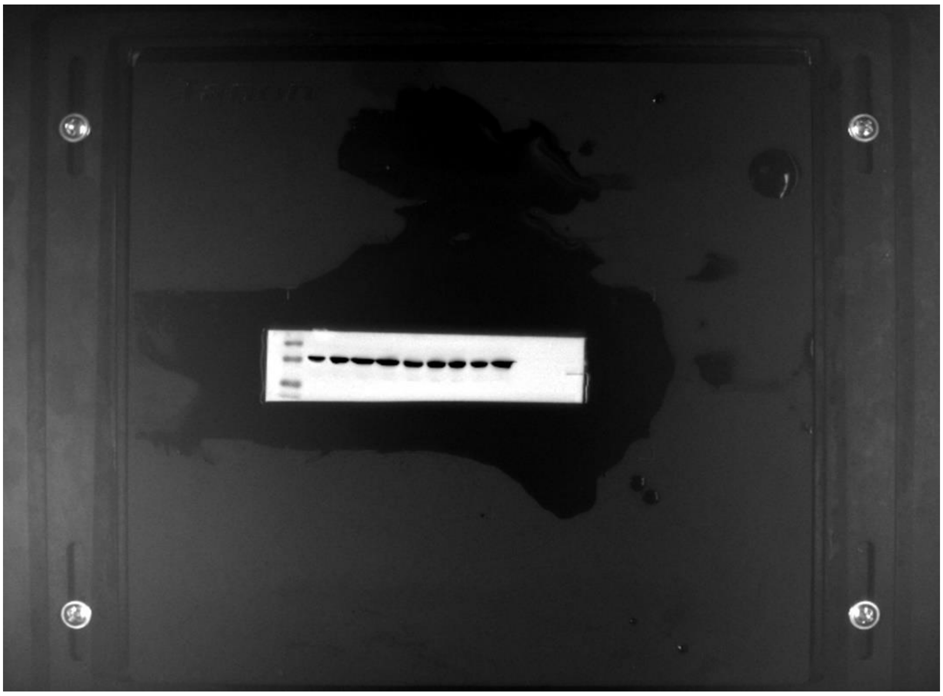

SLC7A11

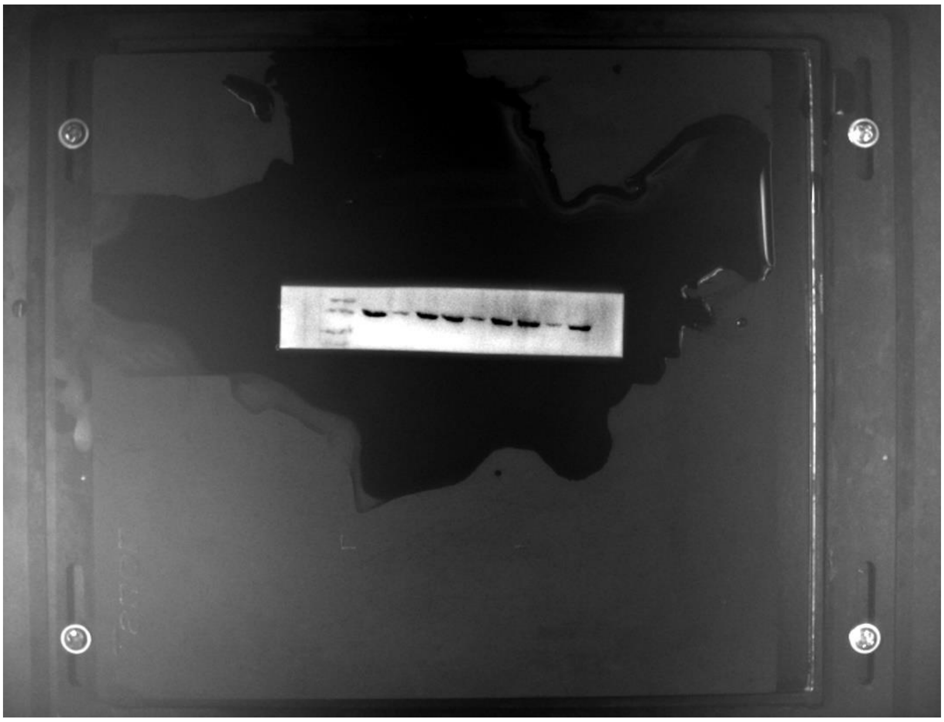

Fig 9G

β -actin

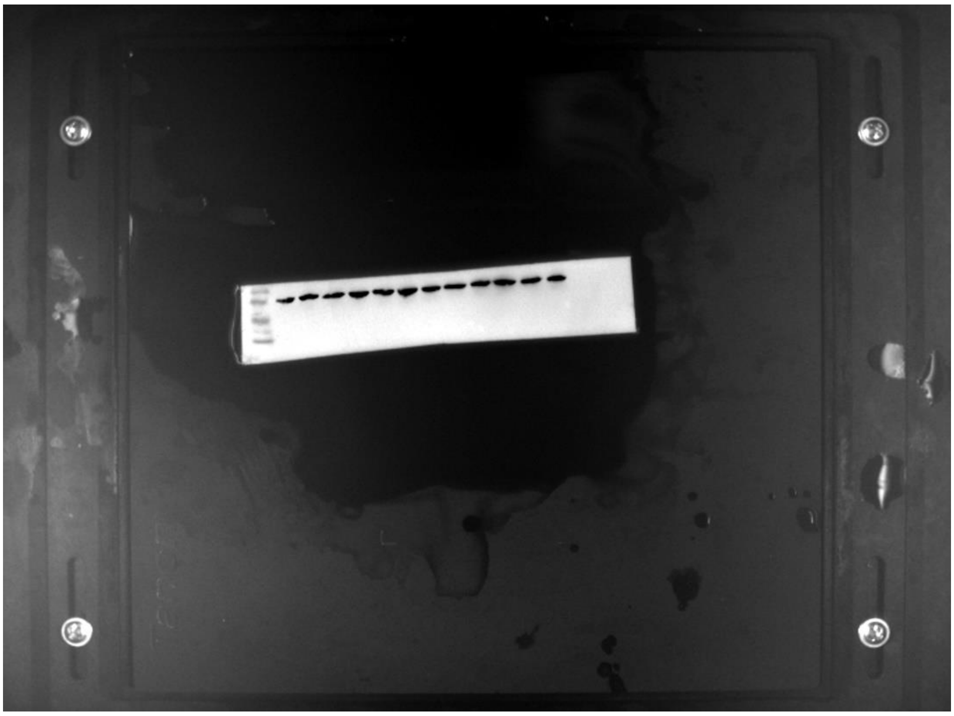

SLC7A11

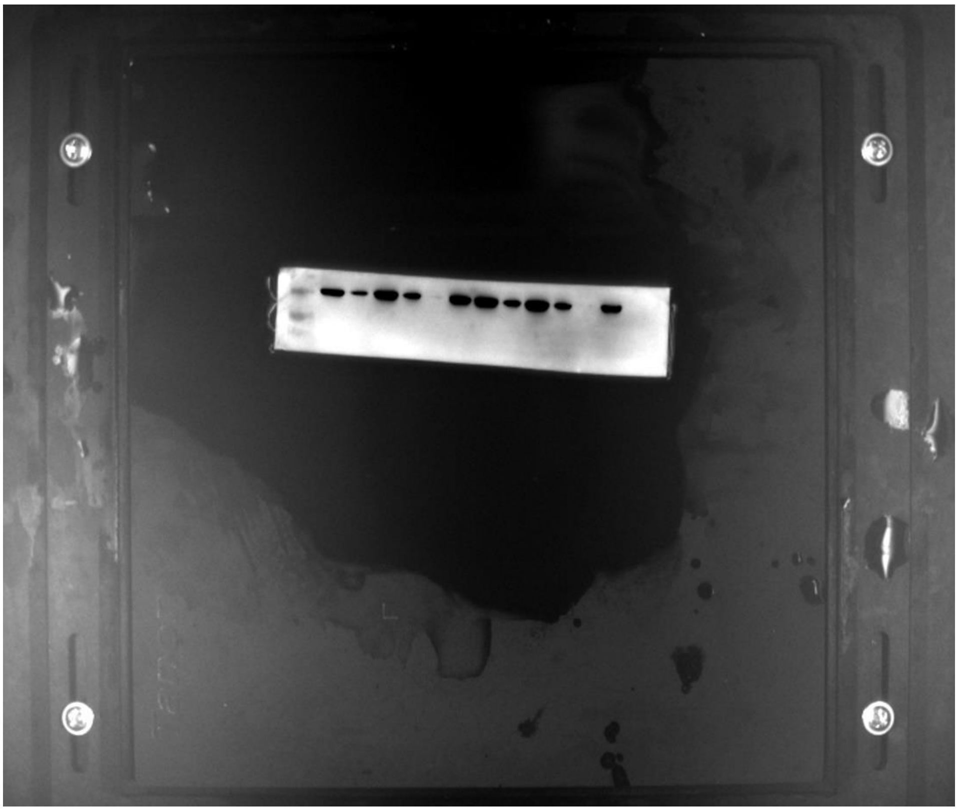

Fig 9G

GPX4

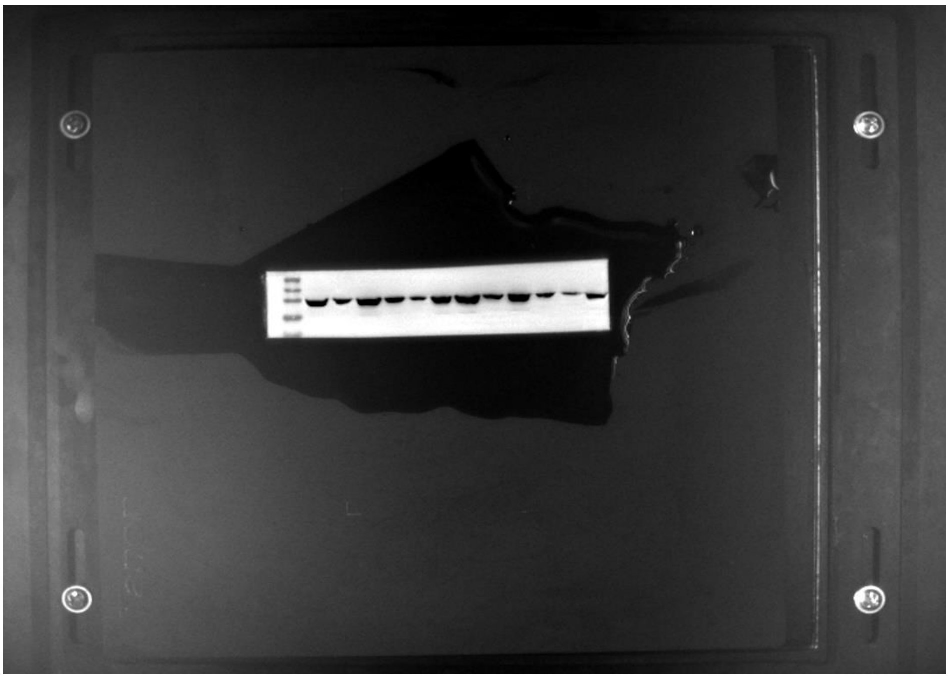

TXNIP

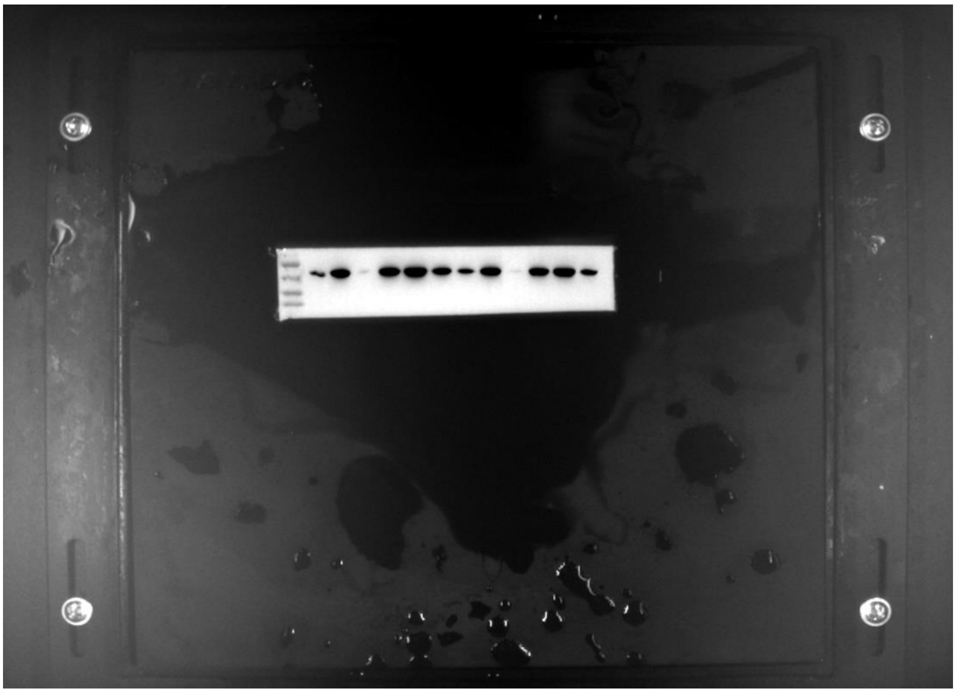

Supplement: Supplementary file 1 — Supplementary Material 1 [file 41598_2025_19092_MOESM1_ESM.pdf]
